# Caffeic acid phenethyl ester protects renal tubular epithelial cells against ferroptosis in diabetic kidney disease via restoring PINK1-mediated mitophagy

**DOI:** 10.1186/s10020-025-01318-y

**Published:** 2025-07-24

**Authors:** Ying Lu, Ye Zhu, Sheng Feng, Qifei Cong, Sixia Chen, Ying Zeng, Kai Song, Ji Hu

**Affiliations:** 1 Department of Nephrology, The Second Affiliated Hospital of Soochow University, Suzhou, 215004 China; 2 Institute of Neuroscience and Jiangsu Key Laboratory of Neuropsychiatric Diseases, Soochow University, Suzhou, 215123 China; 3 Department of Nursing, The Second Affiliated Hospital of Soochow University, Suzhou, 215004 China; 4 Department of Endocrinology, The Second Affiliated Hospital of Soochow University, Suzhou, 215004 China

**Keywords:** Caffeic acid phenethyl ester, Diabetic kidney disease, Tubular epithelial cells, Ferroptosis, Mitophagy

## Abstract

Mounting evidence indicates that renal tubular ferroptosis plays a crucial role in the progression of diabetic kidney disease (DKD). Caffeic acid phenethyl ester (CAPE), derived from propolis, a precious resinous substance synthesized by various bee species, has garnered broad attention in biomedical research. This study aims to explore the mechanism by which CAPE protects renal tubular epithelial cells (TECs) against ferroptosis in DKD. DBA/2J mice were administered streptozotocin (STZ) by intraperitoneal injection, fed a high-fat diet (HFD) and treated with CAPE. The findings revealed significant changes in ferroptosis markers. In diabetic mice and TECs under high-glucose (HG) conditions, levels of glutathione peroxidase 4 (GPX4) and solute carrier family 7 member 11 (SLC7A11) decreased, while transferrin receptor 1 (TFR1) increased. These changes were accompanied by a reduction in antioxidant capability and the accumulation of malondialdehyde (MDA). Gene Ontology (GO) and Kyoto Encyclopedia of Genes and Genomes (KEGG) enrichment analyses showed that the intersection targets of CAPE and ferroptosis were mainly located in the mitochondria and exhibited high enrichment values in mitophagy. Further investigations revealed that HG induced a depolarization of mitochondrial membrane potential and an excessive level of mitochondrial reactive oxygen species (ROS), accompanied by defective mitophagy. The administration of CAPE inhibited exacerbated ferroptosis and rescued defective mitophagy induced by DKD. In addition, CAPE restored PTEN-induced putative kinase 1 (PINK1) levels, which were markedly diminished in the kidneys of DKD mice and TECs subjected to HG. Molecular docking simulation experiments suggested that CAPE is steadily bound to the PINK1 active pocket. Cellular Thermal Shift Assay (CETSA) and Drug Affinity Responsive Target Stability assay (DARTS) showed that CAPE enhances the thermal stability of the PINK1 protein within a specific temperature range and protects the PINK1 protein from degradation by proteolytic enzymes. These results confirm that CAPE interacts with PINK1 as its specific target. However, the positive outcomes of CAPE treatment on ferroptosis were nullified by the PINK1 siRNA. This research indicates that CAPE has potential therapeutic benefits for DKD by protecting renal TECs against ferroptosis via rescuing PINK1-mediated mitophagy. These findings suggest that CAPE shows potential as a therapeutic agent to prevent tubular injury in DKD.

**Supplementary Information:**

The online version contains supplementary material available at 10.1186/s10020-025-01318-y.

## Introduction

Diabetic kidney disease (DKD), a leading cause of chronic renal failure, stands as one of the most severe complications of diabetes mellitus (DM) [1, 2]. Conventionally, glomerular pathology has been regarded as the primary focus of DKD. However, emerging research has shed light on the fact that tubular damage plays a pivotal role in the deterioration of renal function [3–5]. Consequently, preserving normal renal tubular function is crucial for decelerating the progression of DKD.

Ferroptosis, a distinct form of cell death characterized by iron-dependent lipid peroxidation, represents a non-apoptotic and regulated mode of cell demise [6, 7]. Recently, accumulating evidence has revealed a close association between the ferroptosis of tubular epithelial cells (TECs) and DKD. The key mediators of ferroptosis, glutathione peroxidase 4 (GPX4) and solute carrier family 7 member 11 (SLC7A11), are present at significantly lower levels in the renal tubules of DKD patients compared to non-diabetic individuals [8, 9]. Ferroptosis inducers have been shown to trigger the death of TECs [10]. In contrast, the addition of ferrostatin-1 to DKD models has significantly upregulated SLC7A11 and GPX4 levels [11]. Simultaneously, it has alleviated lipid peroxidation and renal pathological damage [11]. Furthermore, when DKD mice or renal tubular epithelial cells (HK-2 cells) are treated with Empagliflozin, Dapagliflozin, or Semaglutide, high-glucose (HG)-induced ferroptosis can be mitigated. This is evidenced by a reversal of reduced GPX4 and SLC7A11 expressions in tubular epithelial cells, thereby alleviating diabetic kidney injury [9, 12, 13]. Collectively, the above-mentioned research findings corroborate that renal tubular ferroptosis is a critical aspect of DKD. As a result, targeting the ferroptosis of TECs emerges as an innovative strategy for halting the progression of DKD.

Mitochondrial iron accounts for 20–50% of the total intracellular iron [14, 15]. Besides its involvement in the biosynthesis of iron-sulfur cluster and heme [16, 17], mitochondrial iron also actively promotes mitochondrial ROS generation [18]. The TECs possess the highest mitochondrial content among all cell types within the kidney and are particularly sensitive to mitochondrial malfunction [19, 20]. In usual situations, damaged mitochondria are cleared through mitophagy [21–23]. When mitophagy is impaired, dysfunctional mitochondria are not cleared, resulting in an overproduction of mitochondrial ROS, which reacts with the polyunsaturated fatty acids of the mitochondrial membrane, bringing about lipid peroxidation [24–26]. The best described pathway for triggering mitophagy involves PTEN-induced kinase 1 (PINK1). A prior study showed that MitoQ, a mitochondria-targeted antioxidant, may be effective in reducing tubular damage in DKD by activating PINK1-mediated mitophagy [27]. In addition, mitochondrial morphology exhibited characteristic changes of ferroptosis, along with the buildup of mitoROS in renal proximal TECs cultivated under high glucose (HG) conditions [28]. Researchers have also observed that mitophagy alleviates ferroptosis in renal TECs during acute kidney disease [29, 30]. All these observations consistently suggest the engagement of mitochondria in ferroptosis. However, it remains unclear how mitophagy affects ferroptosis in DKD.

Propolis has long been considered to possess medicinal properties [31]. Caffeic acid phenethyl ester (CAPE), one of the main caffeic acid analogues, has excellent antioxidant properties [32–35]. The beneficial effects of CAPE are associated with the alleviation of mitochondrial dysfunction [36–38]. Sorrenti et al. have suggested that CAPE and its derivatives alleviate oxidative stress in mesangial cells of diabetic rats via regulating the Akt/NF-κB/iNOS and HO-1 pathways, thereby improving DKD[39]. However, the effect of CAPE on mitophagy and whether CAPE can reverse tubular damage in DKD by alleviating ferroptosis are not known.

Based on these observations, it was hypothesized that CAPE could protect against DKD by alleviating ferroptosis through rescuing mitophagy. This study aims to find out the impact of CAPE on mice with DKD and confirm its role in regulating mitophagy and ferroptosis in TECs under HG conditions.

## Materials and methods

### Drugs and reagents

CAPE was obtained from Selleck (S7414, Houston, TX, USA) with a purity of ≥ 99.99% after HPLC purification. Streptozotocin (STZ) was obtained from Sigma (S0130, St Louis, MO, USA). Ferrostatin-1 (a specific ferroptosis inhibitor), Erastin (a ferroptosis inducer) and ML385 (a specific Nrf2 inhibitor) were obtained from MedChemExpress (HY-100579, HY-15763 and HY-100523, USA).

### Animal study design

Eight-week-old male DBA/2J mice were purchased from Phenotek Biotech Company. The mice were housed under a 12-hour light/12-hour dark cycle with ad libitum access to food and water. DKD was induced via a combination of intraperitoneal low-dose STZ injections and a high-fat diet (HFD) [40, 41]. Specifically, mice received daily intraperitoneal injections of STZ (50 mg/kg body weight, dissolved in 50 mM sodium citrate buffer, pH 4.5) for five consecutive days. One week after the final STZ injection, blood glucose levels were measured from the tail vein using an Accu-Check glucose meter. Mice with blood glucose levels exceeding 16.7 mmol/L were classified as diabetic and subsequently maintained on the HFD (D12492, Research Diets, New Brunswick, USA). The HFD contained 60% kcal from fat, 20% kcal from protein, and 20% kcal from carbohydrates, while the normal diet (XTC01WC-001, Xietong Bio, China) contained 11.4% kcal from fat, 21.3% kcal from protein, and 67.3% kcal from carbohydrates. Based on previous in vivo studies, the maximum single dose of CAPE administered intraperitoneally was reported to reach 34 mg/kg [42], and multiple dosing regimens typically ranged from 2.84 to 10 mg/kg per day [43, 44]. In our preliminary experiments, three CAPE doses (1, 5, and 10 mg/kg/day) were selected for evaluation. The mice were grouped as follows: (1) Ctrl group: intraperitoneal injection of 50 mM sodium citrate buffer only, normal diet feeding, *n* = 6; (2) CAPE group: intraperitoneal injection of 50 mM sodium citrate buffer only, normal diet feeding, administration of CAPE at 5 mg/kg/day via gastric gavage, *n* = 6; (3) DM group: received intraperitoneal injection of STZ and were fed a high-fat diet, *n* = 6; (4) DM + CAPE group: received intraperitoneal injection of STZ and were fed a high-fat diet, and were administered CAPE at 1, 5 and 10 mg/kg/day, respectively, via gastric gavage from one week after STZ injection to week 18, *n* = 6.

At the end of the 18-week study, blood samples were collected from the retro-orbital venous plexus under isoflurane anesthesia for biochemical parameter analysis. Subsequently, euthanasia was humanely performed via cervical dislocation. Urine was aspirated via sterile bladder puncture to measure the urinary microalbumin, urinary creatinine, and renal injury biomarkers. Kidneys were rapidly excised. The left kidney was fixed in 4% paraformaldehyde for histopathological examination, while the right renal cortex was dissected on ice and snap-frozen in liquid nitrogen for subsequent protein extraction and immunoblotting analysis. The study followed the principles outlined in the ARRIVE Guidelines and obtained approval from the Ethics Committee for Experimental Animal Care and Use at the Second Affiliated Hospital of Soochow University (EC2024339). No animal mortality was observed throughout the experimental period.

### Sampling of kidney samples

Formalin-fixed paraffin-embedded specimens were collected from patients diagnosed with DKD at the Second Affiliated Hospital of Soochow University (DKD1 group: DKD patients with an estimated glomerular filtration rate (eGFR) ≥ 60 ml/min, *n* = 5; DKD2 group: DKD patients with an eGFR < 60 ml/min, *n* = 5) and from the para-cancerous tissues after nephrectomy (Control group: patients with a GFR ≥ 60 ml/min, *n* = 5). The eGFR is obtained through the CKD-EPI equation. Renal tissues were stained with periodic acid-Schiff (PAS) and Masson’s trichrome staining. The study was conducted in accordance with the principles of the Helsinki Declaration and obtained approval from the Medical Ethics Committee of the Second Affiliated Hospital of Soochow University (JD-HG-2023-67), and informed consent was obtained from all patients.

### Biochemical analyses of blood and urine

Urea nitrogen (UN) in the serum and urine creatinine levels were determined using enzymatic methods by the clinical laboratory of the Second Affiliated Hospital of Soochow University. Urine albumin was detected using an ELISA quantitative assay kit (E99-134, Bethyl Laboratory, Houston, TX). Subsequently, the urinary albumin/creatinine ratio (UACR) was calculated. KIM-1 (LV30344, Animaluni, Shanghai, China) and 8-OHdG (LV30717, Animaluni, Shanghai, China) in urine were measured using ELISA kits.

### Histological examination

The glomerular tuft area was assessed using Periodic Acid–Schiff (PAS) staining. The collagen-positive area was assessed by Picrosirius Red staining. The glomerular tuft area was measured using Slide Viewer software. Interstitial collagen was assessed in a blinded fashion using Image J 1.8.0. The results were expressed as the average of 10 independent measurements per slide.

### Immunohistochemical examination

An immunohistochemical examination was performed on 5 μm formalin-fixed kidney sections. After routine deparaffinization, the slices were treated with 3% H2O2 to eliminate endogenous peroxidase activity and were followed by antigen retrieval using proteinase K. The slides were blocked with 10% donkey serum. Primary antibodies were added to the slides. Specifically, these included antibodies against CD68 (1:200 dilution, GB113109, Servicebio, Wuhan, China), E-Cadherin (E-Cad) (1:100 dilution, #3195, Cell Signaling Technology, USA), GPX4 (1:200 dilution, 67763-1-Ig, Proteintech, Wuhan, China), SLC7A11 (1:200 dilution, 26864-1-AP, Proteintech, Wuhan, China), PINK1 (1:200 dilution, 23274-1-AP, Proteintech, Wuhan, China), Parkin (1:100 dilution, ab77924, Abcam, UK), P62 (1:200 dilution, ab109012, Abcam, UK), TFR1 (1:100 dilution, ab214039, Abcam, UK) and LC3B (1:200 dilution, ab192890, Abcam, UK). At least 5 fields of view were examined for each slide using a microscope (Zeiss Axio Scope A1, Germany). Analysis was conducted using Image J 1.8.0, and the results were represented as the average of five independent measurements per slide.

### Western blot assay

The western blot was performed using total cellular proteins, cytoplasmic proteins, and nuclear proteins. Total proteins were extracted from tissues or cells using RIPA buffer (Radioimmunoprecipitation Assay, containing 1× protease inhibitor cocktail, 1× PMSF, and 1× phosphatase inhibitor). Both tissue and cell lysates were incubated on ice for 10 min to ensure complete lysis, followed by centrifugation at 12,000 ×g for 15 min at 4 °C. The supernatant was collected as the total protein extract. Cytoplasmic and nuclear proteins were separated using the NE-PER™ Nuclear and Cytoplasmic Extraction Kit (78833, Thermo Fisher Scientific, USA). Briefly, cells were suspended in cytoplasmic extraction reagent, incubated on ice for 15 min, and centrifuged at 12,000 ×g for 10 min at 4 °C. The supernatant (cytoplasmic fraction) was collected. The pellet was resuspended in nuclear extraction reagent, vortexed, incubated on ice for 30 min, and centrifuged at 12,000 ×g for 15 min at 4 °C. The resulting supernatant (nuclear fraction) was collected. A total of 20 µg protein was loaded and separated on SDS-PAGE. Primary antibodies targeting GPX4 (diluted at 1:2000, 67763-1-Ig), PINK1 (diluted at 1:300, 23274-1-AP), GAPDH (diluted at 1:2000, 60004-1-Ig) and SLC7A11 (diluted at 1:1000, 26864-1-AP) were obtained from Proteintech, and antibodies targeting TFR1 (diluted at 1:500, ab214039), LC3B (diluted at 1:2000, ab192890), Parkin (diluted at 1:500, ab77924) and P62 (diluted at 1:1000, ab109012) were obtained from Abcam. A primary antibody targeting Nrf2 (diluted at 1:1000, AF7623) was obtained from Beyotime. Subsequently, the membranes were rinsed and then incubated with the secondary antibody before being developed using an ECL kit (HY - K1005, MedChemExpress, USA). Band intensity was analyzed using Image J 1.8.0.

### Cell culture

HK-2 cells were obtained from Procell Life Science & Technology and cultured in a cell-specific culture medium (CM-0109, Procell, Wuhan, China). We tested a concentration range of 1–20 µM CAPE and determined suitable doses for subsequent experiments through cell viability assays. The HK-2 cells were grouped as follows: Ctrl group, which was cultured in 5.5 mM glucose medium; CAPE group, which was cultured in 5.5 mM glucose medium containing CAPE; HG group, which was cultured in 30 mM glucose medium; HG + CAPE group, which was cultured in 30 mM glucose medium containing CAPE; Fer-1 group, which was cultured in 5.5 mM glucose medium with 1 µM ferrostatin-1; HG + Fer-1 group, which was cultured in 30 mM glucose medium with 1 µM ferrostatin-1. ML385 is a specific inhibitor of Nrf2. We used ML385 (2 µM) to evaluate whether the inhibitory effect of CAPE on ferroptosis was achieved through the activation of the Nrf2 pathway.

### Cell viability determination

The cells viability was assessed using the CCK-8 assay kit (MA0218, Meilunbio, China). Briefly, cells were cultured under the specified conditions as follows. In each well, 10 µl of CCK-8 were added, and then the wells were cultured in an incubator for 2 h. The absorbance was measured at 450 nm using a multifunctional microplate reader.

### Lipid peroxidation evaluation

The iron concentrations were determined using a detection kit (MAK025, Sigma-Aldrich, USA). MDA and superoxide dismutase (SOD) in tissues and cells were detected using the MDA kit (S0131) and SOD kit (S0101), respectively, from Beyotime, Shanghai, China. Glutathione (GSH) in kidneys and cells was assessed using a Reduced GSH kit (EEA020, Invitrogen, USA). The detection procedures were meticulously performed in strict accordance with the manufacturer’s instructions.

The fluorescent probe Liperfluo (L248, Dojindo, Japan) was used to detect lipid hydroperoxides. First, cells were precisely plated in µ-slide plates (ibidi, Germany) and then washed once with serum-free MEM medium to remove any potential contaminants. Next, serum-free medium containing exactly 10 µM Liperfluo was added, and the cells were incubated for 30 min to allow the probe to incorporate into the cells. Subsequently, the cells were washed with HBSS solution to eliminate unbound Liperfluo. Then, the cells were uniformly treated with precisely 200 µl of a solution containing exactly 500 µM tert-Butyl Hydroperoxide (t-BHP) to induce lipid peroxidation. The cells were then placed in an incubator for 30 min to ensure the reaction proceeded. Finally, the fluorescence microscope, set at an excitation wavelength of 488 nm, was utilized to visualize the fluorescent signal of Liperfluo, thereby enabling the detection of lipid hydroperoxides generated within the cells.

### Collection of the targets of CAPE and function analysis

CAPE (CID: 5281787) was searched for in PubChem, SwissTarget Prediction, SuperPred, SEA and PharmMapper databases to collect its targets. Ferroptosis target genes were collected from the FerrDb V2 database. The intersection targets of CAPE and ferroptosis were obtained using Venny 2.1. GO functional and KEGG pathway enrichment analyses were performed on the collected genes using the David database.

### Mitochondrial ROS production

To evaluate the production of mitochondrial ROS, cells were labeled with MitoSox Green (M36007, Invitrogen, USA). Following staining for 30 min at 37 °C, the mitochondrial ROS was visualized using a confocal microscope (Zeiss LSM, Germany).

### Adenosine triphosphate (ATP) assay

Following the guidelines of the enhanced ATP assay kit (S0027, Beyotime, China), ATP levels were measured. The relative light units (RLU) were detected using a multifunctional microplate reader (TECAN Infinite 200 Pro, Switzerland).

### Detection of MMP

The JC-1 Kits (C2003, Beyotime, Shanghai, China) were used to identify mitochondrial membrane potential (MMP). First, the cells were cultured in the working solution for 20 min at 37 °C in a 5% CO₂ incubator. After that, the JC − 1 fluorescence was observed using a confocal microscope (Zeiss, Germany).

### Transmission electron microscopy

The cell aggregates were immersed in a 2.5% glutaraldehyde solution for fixation at 4 °C for 24 h. After the fixation process was completed, the samples underwent dehydration in a series of graded ethanol solutions (30%, 50%, 70%, 90%, and 100%), with each step lasting 15 min. Then, they were embedded in resin at 37 °C. The resin blocks were subsequently sectioned into thin slices of 60–80 nanometers using an ultramicrotome. The slices were then stained on 150-mesh copper grids. Finally, mitochondrial structures were examined under a transmission electron microscope.

### Ad-GFP-LC3B transfection

Ad-GFP-LC3B (C3006, Beyotime, Shanghai, China) was purchased from Beyotime Biotechnology. The cells were incubated with Ad-GFP-LC3B at a multiplicity of infection (MOI) of 10 for 72 h, and then the medium was replaced with the fresh medium. Autophagy in the cells was observed using a confocal microscope (Zeiss LSM, Germany).

### Colocalization of mitochondria and lysosomes

MitoTracker Red CMXRos (C1035, Beyotime, Shanghai, China) was used to stain mitochondria, and Lyso Tracker Green (C1047, Beyotime, Shanghai, China) was used to stain lysosomes. The detailed steps were as follows: After the cell culture medium was carefully aspirated, 100 µL of Mito Tracker Red CMXRos staining working solution with a concentration of 10 µM and 100 µL of Lyso Tracker Green staining working solution with a concentration of 5 µM were added to the cells. Thirty minutes later, the staining working solutions were gently removed. Subsequently, the cells were visualized using a confocal microscope (Zeiss LSM, Germany) with an excitation wavelength of 579 nm for Mito Tracker Red CMXRos and 494 nm for Lyso Tracker Green, and an emission wavelength of 599 nm for Mito Tracker Red CMXRos and 517 nm for Lyso Tracker Green.

### Molecular docking

#### Preparing protein 

The AlphaFold2 was employed to construct the full-length structure of PINK1 protein according to its sequence. Subsequently, the structure was processed using PyMOL software to remove water molecules through a combination of atomic type and coordinate recognition and hydrogen bond network analysis. The resulting structure was imported into AutoDockTools software, where hydrogenation, charge calculation by Charges Compute Gasteiger, and atom types were sequentially assigned, and ultimately output the structure in the software-specific.pdbqt format. The specific steps are as follows: Add hydrogen atoms: Select “Edit"→"Hydrogens"→"Add”. We chose “Polar Only” to add only polar hydrogen atoms, and clicked “OK” to complete the hydrogen addition operation. The software automatically calculated the Gasteiger charges of each atom in PINK1 and assigned charge values, and assigned the atom types used in AutoDock to each atom based on the molecular structure, the elemental information of the atoms, and the previously calculated charge information.

#### Preparing receptor molecule

The CAPE (CAS104594-70-9) SDF format structure file was downloaded from the PubChem database. Initially, the CAPE structure was optimized using Chemdraw 3D software. Subsequently, AutoDockTools software was utilized for hydrogenation, calculating the maximum number of rotatable bonds, and setting rotatable bonds. The final step involved exporting the structure in the CAPE.pdbqt format.

#### Molecular docking

Molecular docking was conducted using the internal Vina module within the PyRx software (https://pyrx.sourceforge.io/). The size of the grid box was adjusted according to the spatial range of the active site to ensure that it could completely cover the key areas interacting with the ligand. Specifically, during the present calculation, the docking center was (x = 47.043Å, y = 43.733Å, z = 7.645 Å), and the dimensions of the grid box in x, y, and z directions were all 66.15 Å.

#### Results analysis

The three-dimensional structure and electrostatic potential were visualized using PyMOL. Additionally, two-dimensional structures were visualized using LigPlus.

### Drug affinity responsive target stability (DARTS)

Based on the principle that the binding of small-molecule drugs to target proteins reduces the sensitivity of target proteins to proteases, the DARTS assay was conducted to verify whether CAPE binds to PINK1. The DARTS assay was performed following the method described in reference[45]. Cells were lysed in M- PER (78501, Thermo Fisher Scientific). Subsequently, a 10-fold concentrated TNC buffer (50 mM Tris-HCl, pH 8.0; 50 mM NaCl; 10 mM CaCl₂) was added to the cell lysate. The protein concentration was determined using the Bicinchoninic Acid (BCA) method. Each sample was then incubated with CAPE (200 µM) or DMSO at room temperature for 1 h, followed by digestion with different concentrations of thermolysin (T7902, Sigma) at room temperature for 10 min. After digestion, to terminate the proteolysis reaction, 0.5 M EDTA (pH 8.0) was added to each sample at a 1:10 ratio, and the samples were placed on ice. The samples were then separated by SDS-PAGE. Western blotting was performed to detect whether PINK1 was a direct target of CAPE, with GAPDH used as an internal reference.

### Cellular thermal shift assay (CETSA)

Based on the principle that the binding of small-molecule drugs to target proteins increases the thermal stability of target proteins, the Cellular Thermal Shift Assay (CETSA) was carried out to verify whether CAPE binds to PINK1. The steps before protease digestion were identical to those in the DARTS assay. Instead of protease treatment, each sample was incubated at different temperatures for 3 min. Subsequently, all samples were centrifuged at 20,000 g at 4 °C for 20 min. The precipitate was discarded, and the supernatant was used to detect PINK1 by Western blotting.

### Immunofluorescence staining

HK-2 cells were seeded onto coverslips and cultured to 70–80% confluence. The culture medium was aspirated, and the cells were washed three times with PBS, 5 min each time. Subsequently, the cells were fixed with 4% paraformaldehyde at room temperature for 30 min. After fixation, the cells were washed again with PBS three times, 5 min each time. To permeabilize the cells, 0.1% Triton X − 100 was applied for 10 min. Then, the cells were blocked with 5% bovine serum albumin (BSA) for 2 h. The primary antibody, anti-Parkin (phospho S65) (diluted 1:500, ab315377) or anti-Nrf2 (diluted at 1:1000, AF7623, Beyotime, China) was added to the cells and incubated at 4 °C overnight. After thorough washing with PBS, the Cy3-labeled goat anti-rabbit IgG (H + L) (diluted 1:500, A0516, Beyotime, China) was added. In order to study the colocalization of Parkin with mitochondria, Mito-Tracker Green (diluted to 200nM, C1048, Beyotime, China) was added. The samples were incubated in the dark at room temperature for 1 h. Following the incubation, the coverslips were carefully mounted onto glass slides using an anti-fluorescence quenching mounting medium (P0131, Beyotime, China). The fluorescence signals were visualized using a confocal microscope (Zeiss LSM, Germany). The colocalization of Parkin and Mito-Tracker Green was quantitatively analyzed using Image J 1.8.0 software.

### SiRNA transient transfection

Negative control siRNA (sicontrol) or siRNA targeting PINK1 was transfected (GenePharma, Shanghai, China) into cells by Lipofectamine^TM^2000 as previously described [46] (11668, Invitrogen, Carlsbad, CA). PINK1 siRNA oligonucleotides were as follows: PINK1 siRNA: sense 5′- GAAGCCAUCUUGAACACAATT-3′, antisense 5′-UUGUGUUCAAGAUGGCUU CTT-3′.

### Statistical analysis

Statistical analyses were performed using GraphPad Prism 8.0. Data are expressed as mean ± standard deviation (SD). Comparisons between two groups were analyzed using a two-tailed *Student’s t-test*. For comparisons involving three or more groups, one-way analysis of variance (ANOVA) was applied, followed by *post hoc* Tukey’s test for multiple comparisons. A *p*-value < 0.05 was considered statistically significant.

## Results

### CAPE improved the biochemical parameters and alleviated the glomerular pathological changes in diabetic mice

**Fig. 1:**
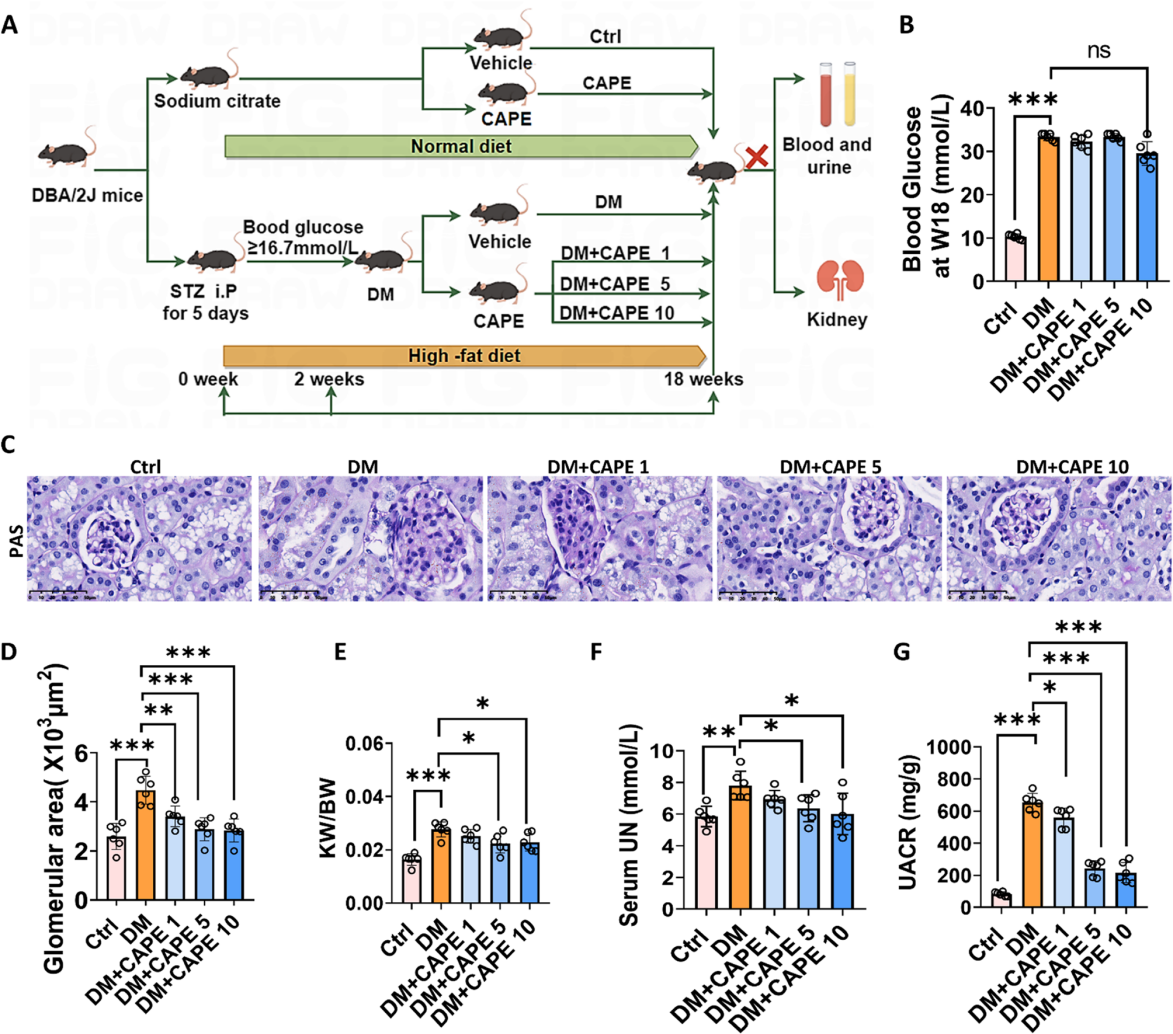
CAPE improved biochemical parameters and alleviated glomerular pathological changes in diabetic mice. **A** Schematic diagram of STZ/HFD-induced T2DM and CAPE intervention. Mice in the DM group received intraperitoneal streptozotocin (STZ) injections and were fed a high-fat diet (HFD), while mice in the DM + CAPE group were administered CAPE (1, 5, or 10 mg/kg/day) by oral gavage starting at week 2. **B** Blood glucose levels. CAPE did not significantly reduce hyperglycemia in diabetic mice. **C** and **D** Representative Periodic acid-Schiff (PAS)-stained kidney sections (Magnification, ×400) and semiquantitative analysis of glomerular area. Diabetic mice exhibited glomerular enlargement compared to controls. **E-G** Kidney weight-to-body weight ratio (KW/BW), serum urea nitrogen (UN), and urinary albumin-to-creatinine ratio (UACR). Diabetic mice showed significant increases in KW/BW, serum UN, and UACR, which were attenuated by CAPE treatment (5 and 10 mg/kg). Data were presented as mean ± SD (*n* = 6). ^*^P < 0.05; ^**^P < 0.01; ^***^P < 0.001; ns, no significance

We established a DKD model in DBA/2J mice by intraperitoneally injecting STZ and feeding a high-fat diet. The experimental procedure was illustrated in Fig. 1A. Compared with the control group, the blood glucose levels in the diabetic mice increased significantly, while CAPE administration had no notable effect on the mice’s blood glucose levels (Fig. 1B). Compared with the control mice, the diabetic mice had enlarged glomeruli (Fig. 1C and D). Additionally, the kidney weight to body weight ratio (KW/BW), serum urea nitrogen (UN), and urinary albumin to creatinine ratio (UACR) in the diabetic mice increased significantly **(**Fig. 1E-G). CAPE treatment at 5 and 10 mg/kg/day significantly attenuated the above-mentioned indexes in diabetic mice (Fig. 1C-G).

### CAPE attenuated the renal tubular injury in diabetic mice

**Fig. 2:**
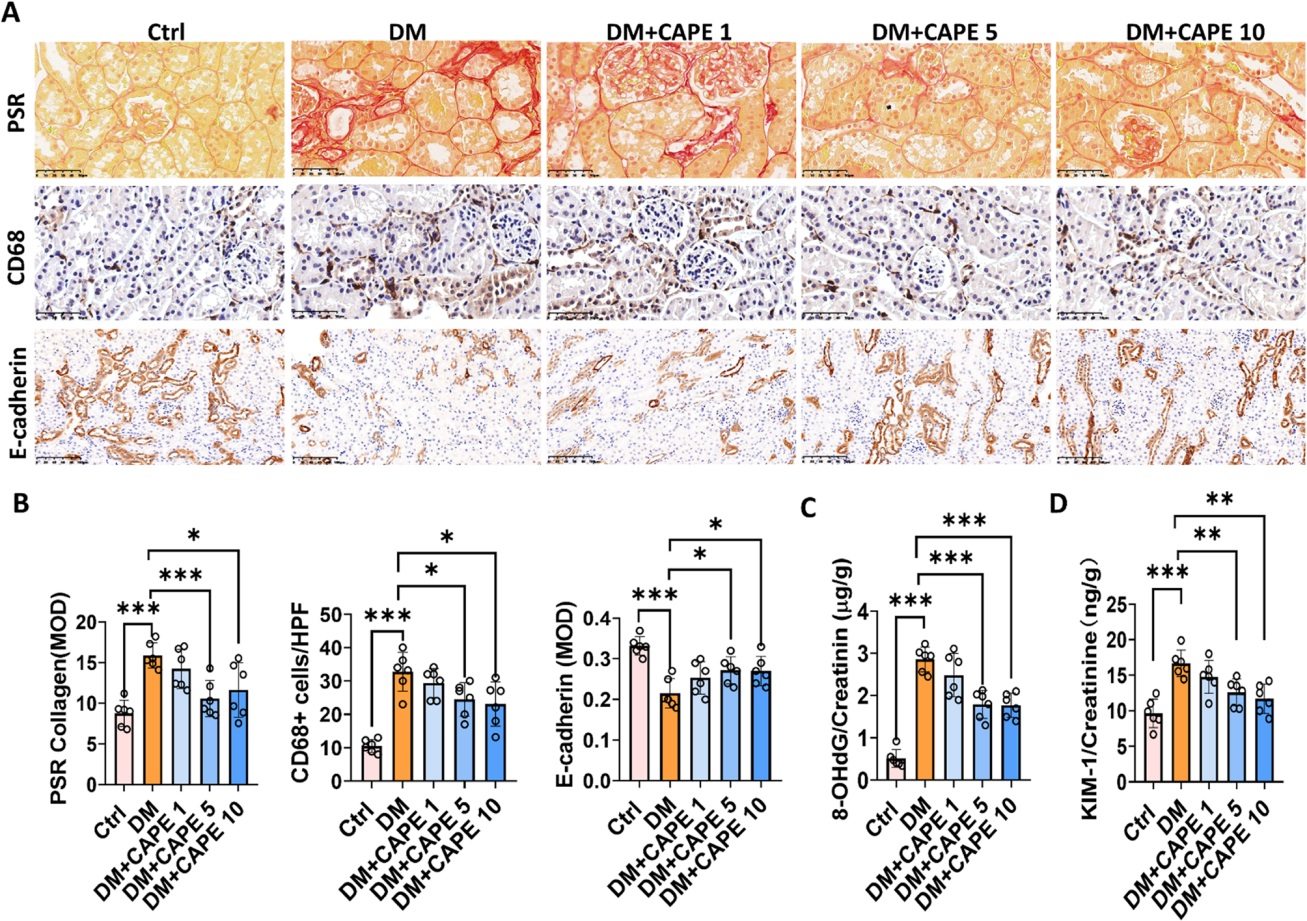
CAPE mitigated the renal tubular injury in diabetic mice. **A** The representative kidney sections stained with picrosirius red, and those stained for CD68 (Magnification, ×400) and E-cadherin (Magnification, ×200). **B** Semi-quantification of interstitial collagen deposition and the results of IHC staining of CD68 + cells and E-cadherin shown in the images. Diabetic mice exhibited increased interstitial fibrosis, elevated CD68 + cell infiltration, and reduced tubular E-cadherin expression, all of which were reversed by CAPE at 5 and 10 mg/kg. **C **and **D** Urinary 8-OHdG/creatinine and KIM-1/creatinine ratios in diabetic mice. On the final day of the 18th week, urine samples were collected from all experimental mice. Urinary levels of 8-OHdG, KIM-1, and creatinine were quantified using standardized assays. Biomarker concentrations were normalized by calculating the 8-OHdG/creatinine and KIM-1/creatinine ratios. CAPE administration significantly reduced the normalized ratios of these tubular injury biomarkers. Data were presented as mean ± SD (*n* = 6). ^*^P < 0.05; ^**^P < 0.01; ^***^P < 0.001

To evaluate the renal tubular damage, the degree of renal interstitial fibrosis, the extent of inflammatory cell infiltration, and the expression of the tubular marker protein E-cadherin were detected. The results showed that compared with the control mice, the DM mice had significantly increased the Sirius red positive area and the number of CD68 positive cells in the renal interstitium and significantly decreased the expression of E-cadherin in the renal tubules (Fig. 2A and B). Compared with the DM group, CAPE treatment at 5 and 10 mg/kg/day alleviated renal interstitial fibrosis, reduced inflammatory cell infiltration, and significantly restored the expression of E-cadherin in the renal tubules of the mice (Fig. 2A and B). Compared with the control mice, the diabetic mice showed significantly higher levels of 8-OHdG and KIM-1 in urine (Fig. 2C and D). Treatment with CAPE at doses of 5 and 10 mg/kg/day markedly reduced the levels of urinary 8-OHdG and KIM-1 in diabetic mice, indicating that CAPE may mitigate renal tubular injury (Fig. 2C and D). Thus, CAPE at 5 mg/kg/day can significantly reduce renal injury in diabetic mice, so we used this dose for future experiments.

### CAPE attenuated ferroptosis in diabetic mice

**Fig. 3:**
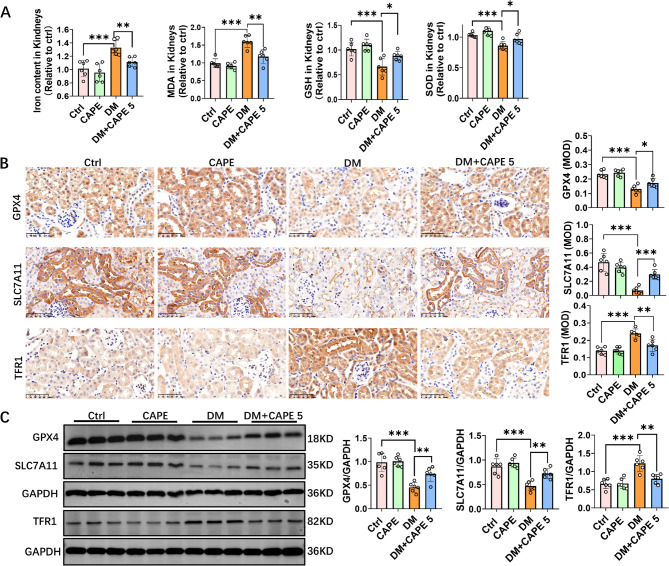
CAPE attenuated ferroptosis in diabetic mice. **A** The renal iron content, malondialdehyde (MDA), reduced glutathione (GSH), and superoxide dismutase (SOD) levels in kidney tissues were quantified in different groups of mice. Untreated diabetic mice exhibited significantly elevated renal iron content and MDA levels in kidney tissues, accompanied by a significant reduction in GSH and SOD levels. Following CAPE treatment, there was partial alleviation of the alterations. **B** Immunohistochemical staining of GPX4, SLC7A11, and TFR1 in the kidney tissues of mice in each group (Magnification, ×400) **(C)** Western blot analysis of the expression of GPX4, SLC7A11, and TFR1 in kidney tissues of different mice. Diabetic mice exhibited decreased GPX4, SLC7A11 and increased TFR1, reversed by CAPE. Data were presented as mean ± SD (*n* = 6). ^*^P < 0.05; ^**^P < 0.01; ^***^P < 0.001

Previous research has suggested that ferroptosis is pivotal in tubular damage in DKD [8, 11]. To elucidate the mechanism by which CAPE mitigates renal tubulointerstitial injury, we evaluated the ferroptosis-related markers in kidney tissues. In contrast to the control group, the untreated DM mice showed a marked elevation in iron content and MDA levels, but a significant reduction of GSH and SOD levels, which indicated enhanced lipid oxidation damage in renal tissues (Fig. 3A). Treatment of DM mice with CAPE significantly reduced the iron content and MDA levels in kidney tissues, while upregulating GSH and SOD levels (Fig. 3A). GPX4 is acknowledged as a pivotal enzyme involved in the suppression of ferroptosis and SLC7A11 is a crucial amino acid transporter that suppresses ferroptosis. Immunohistochemical and immunoblot analysis showed that GPX4 and SLC7A11 were markedly reduced in the DM mice (Fig. 3B and C). TFR1 is a membrane protein receptor responsible for iron complex endocytosis and is markedly increased in the DM mice (Fig. 3B and C). CAPE treatment upregulated GPX4 and SLC7A11, while downregulating TFR1 (Fig. 3B and C). Overall, these results suggest that CAPE can inhibit ferroptosis in the kidney tissues of DM mice.

### CAPE attenuated ferroptosis in HG-challenged TECs

**Fig. 4:**
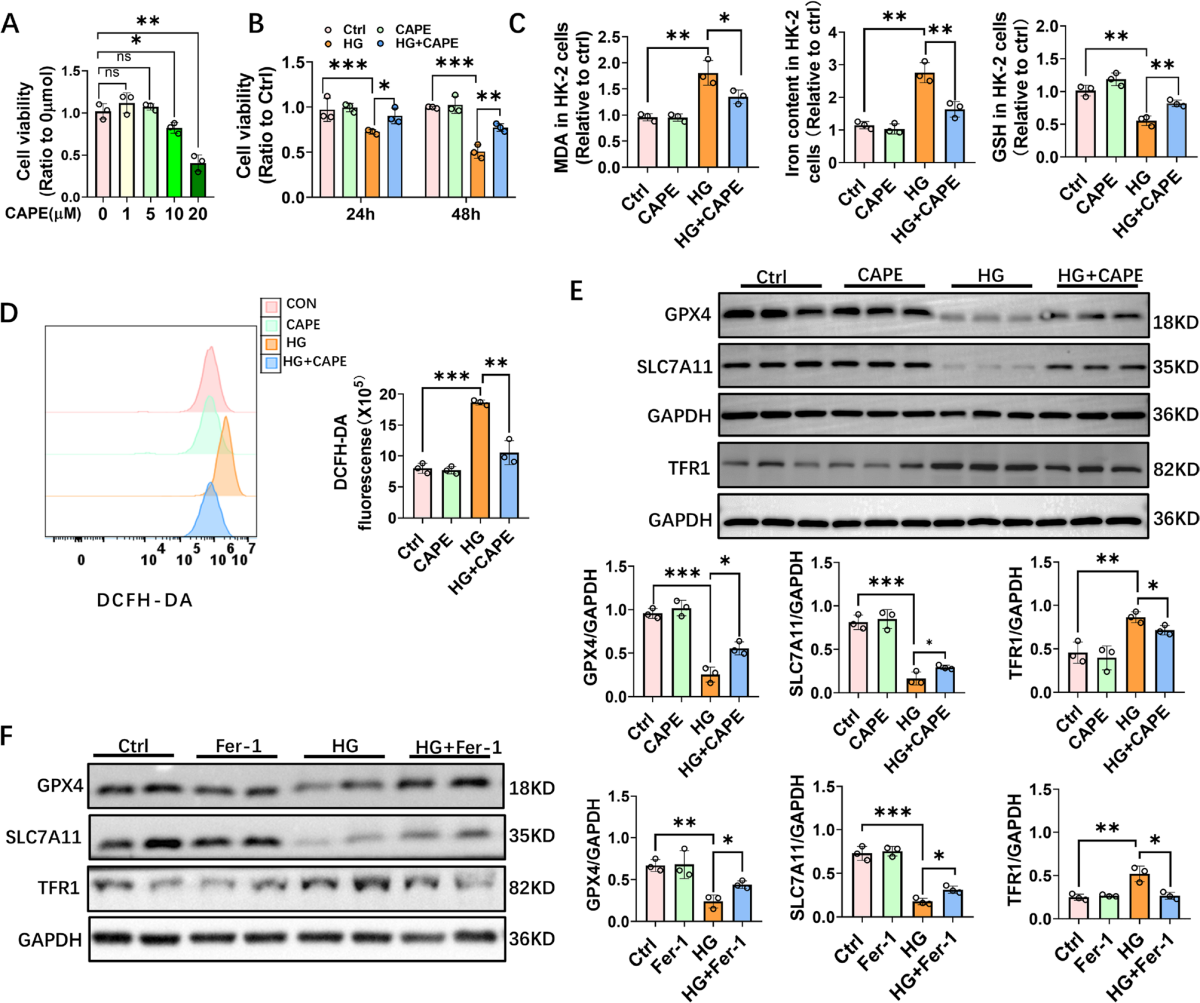
CAPE attenuated ferroptosis in HG-challenged HK-2 cells. **A** Viability of HK-2 cells was measured by CCK-8 assay. The results indicated that 5 µM CAPE had no significant effect on HK-2 cell viability, whereas 10 and 20 µM CAPE caused significant cytotoxicity. **B** HK-2 cells were exposed to 30 mM glucose and 5 µM CAPE for 24 and 48 h. CAPE (5 µM) effectively inhibited the cell death induced by high glucose (HG) at both time points. **C** The levels of MDA, iron content, and GSH in HK-2 cells were measured in each experimental group. HG triggered significant lipid peroxidation, iron overload, and reduced antioxidant capacity in HK-2 cells. **D** ROS production in HK-2 cells from each group was visualized by DCFH-DA staining. **E** Western blot analysis of GPX4, SLC7A11, and TFR1 expression in HK-2 cells. HG significantly decreased GPX4 and SLC7A11 levels while increasing TFR1 expression, which were partially reversed by CAPE treatment. **F** The effect of Fer-1 on HG-induced ferroptosis in HK-2 cells. Cells were cultured with 30 mM glucose and 5 µM Fer-1 for 48 h. Fer-1 restored HG-induced reductions in GPX4 and SLC7A11 and reversed TFR1 upregulation. All data are expressed as mean ± SD (*n* = 3). ^*^P < 0.05; ^**^P < 0.01; ^***^P < 0.001

We used varying concentrations of CAPE from 1 to 20 µM and measured cell viability by the Cell Counting Kit-8. When the concentration of CAPE reached 10 µM, the findings indicated a marked reduction in cell viability (Fig. 4A). We further explored whether there was a dose-response dependence by using CAPE concentrations ranging from 0.1 to 10 µM. The results revealed that within the concentration range of 0.1 to 5 µM, as the CAPE dose increased, the inhibitory effect on ferroptosis also increased. However, when compared to the 5 µM dose, the 10 µM dose did not exhibit a more significant advantage in terms of inhibiting ferroptosis. A detailed description of this result can be found in the supplementary figure (Supplementary Fig. 1). Therefore, we used a concentration of 5 µM for further investigations.

Further findings revealed that subjecting HK-2 cells to 30mM glucose for a duration of 24 h led to a significant decline in cell viability, with even lower viability observed at 48 h (Fig. 4B). CAPE (5 µM) inhibited the HG-induced cell death at both 24 h and 48 h (Fig. 4B). The HK-2 cells cultured in HG exhibited significant lipid peroxidation, iron overload, ROS accumulation, and reduced antioxidant capacity (Fig. 4C and D), which are characteristic changes associated with ferroptosis. The underlying mechanism is that these changes are closely related to the dysregulation of the cellular redox balance and iron metabolism, leading to the accumulation of toxic lipid peroxides. However, CAPE notably reversed these changes by potentially modulating the expression and activity of key proteins involved in ferroptosis regulation, such as GPX4 and SLC7A11 (Fig. 4C and D). CAPE and the ferroptosis inhibitor Fer-1 exhibited comparable effects in reversing the reductions of GPX4 and SLC7A11 caused by HG, as well as the elevation of TFR1 (Fig. 4E and F).

### CAPE alleviated mitochondrial injury in HK-2 cells caused by HG

**Fig. 5:**
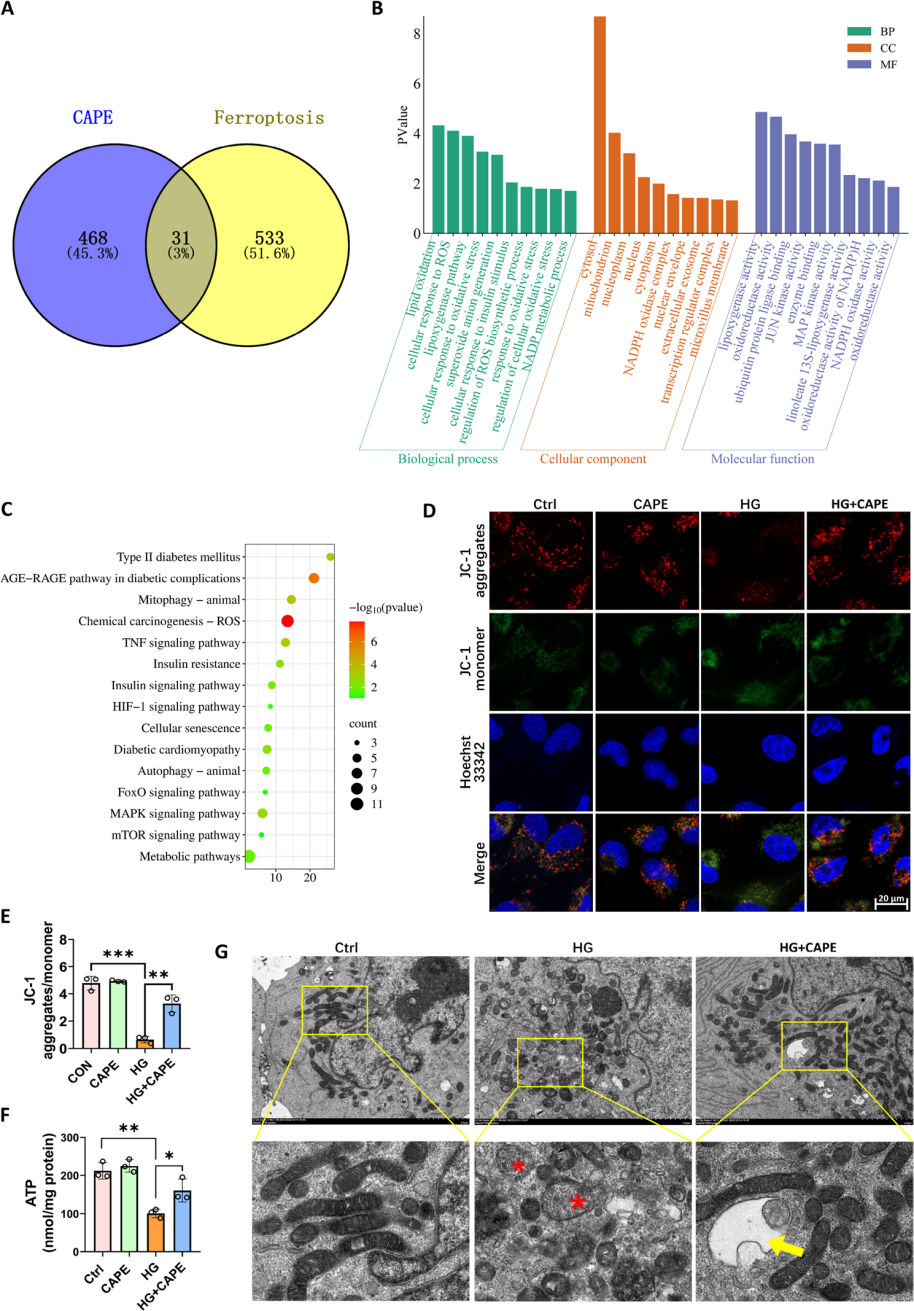
CAPE alleviated HG-induced mitochondrial damage in HK-2 cells. **A** Thirty-one intersection targets of CAPE and ferroptosis. **B** GO enrichment analysis. The intersection targets were primarily localized in the cytosol, mitochondria, nucleoplasm and nucleus. **C** KEGG enrichment analysis. The thirty-one intersection targets were enriched in 88 pathways (*p* < 0.05), including mitophagy and ROS pathways. **D** Mitochondrial membrane potential (MMP) assessed by JC-1 staining in HK-2 cells after 24-hour HG stimulation (scale bar = 20 μm). **E** Quantification of JC-1 aggregate/monomer fluorescence intensity in HK-2 cells. **F** ATP levels in HK-2 cells. HG stimulation for 24 h significantly reduced ATP production, which was reversed by CAPE treatment. **G** Transmission electron microscopy (TEM) images of mitochondria in HK-2 cells (scale bar = 2 μm). Exposure of HK-2 cells to HG for 24 h resulted in mitochondrial shortening accompanied by cristae loss. Asterisks indicate mitochondrial swelling and cristae loss. Arrows indicate autophagosomes. (Magnification, ×7000; High Contrast Mode; Acceleration Voltage 80.0 KV). All data are expressed as mean ± SD (*n* = 3). ^*^P < 0.05; ^**^P < 0.01; ^***^P < 0.001

A total of 499 target genes of CAPE were collected from PubChem, SwissTarget Prediction, SuperPred, SEA and PharmMapper databases. 564 ferroptosis target genes were collected from the FerrDb V2 database. Through Venny 2.1, thirty-one intersection targets of CAPE and ferroptosis were obtained (Fig. 5A). Gene Ontology (GO) enrichment analysis unveiled that the genes were mainly located in the cytosol, mitochondria, nucleoplasm and nucleus (Fig. 5B). Among them, mitochondria ranked second. These genes participate in various biological processes. For example, they are involved in lipid oxidation, the response to ROS and oxidative stress, and the lipoxygenase pathway. They achieve this by modulating molecular functions such as those of lipoxygenase, oxidoreductase, and NADPH oxidase (Fig. 5B). An enrichment analysis of these 31 intersection targets yielded a total of 88 Kyoto Encyclopedia of Genes and Genomes (KEGG) pathways (*p* < 0.05), including the mitophagy and ROS pathways (Fig. 5C). These results suggest that CAPE may affect ferroptosis by regulating the mitophagy and ROS pathways, providing important clues for further research on the mechanism of CAPE on ferroptosis.

The next investigation focused on investigating the impact of HG and CAPE on mitochondria. When the mitochondrial membrane potential (MMP) is normal, JC-1 exists in the mitochondrial matrix as aggregates, resulting in red fluorescence. However, when the MMP was depolarized, JC-1 existed as a monomer and formed green fluorescence. Our results revealed that HG induced the MMP to depolarize at 24 h, which was reversed by CAPE (Fig. 5D and E). Under HG conditions, the ATP produced by HK-2 cells is reduced. CAPE restored the decrease of ATP induced by HG (Fig. 5F). Transmission electron microscopy revealed that the mitochondrial structure of HK-2 cells was significantly changed after 24 h of HG stimulation (Fig. 5G). These changes included shortening of the mitochondrial length, mitochondrial swelling, and loss of mitochondrial cristae. However, CAPE partially restored the mitochondrial morphology. Additionally, although the morphological alterations of mitochondria in HK-2 cells were more pronounced in the HG group, there were fewer autophagosomes observed compared to the Ctrl group and the HG + CAPE group (Fig. 5G).

### CAPE regulated the renal tubular mitophagy in vivo and in vitro

**Fig. 6:**
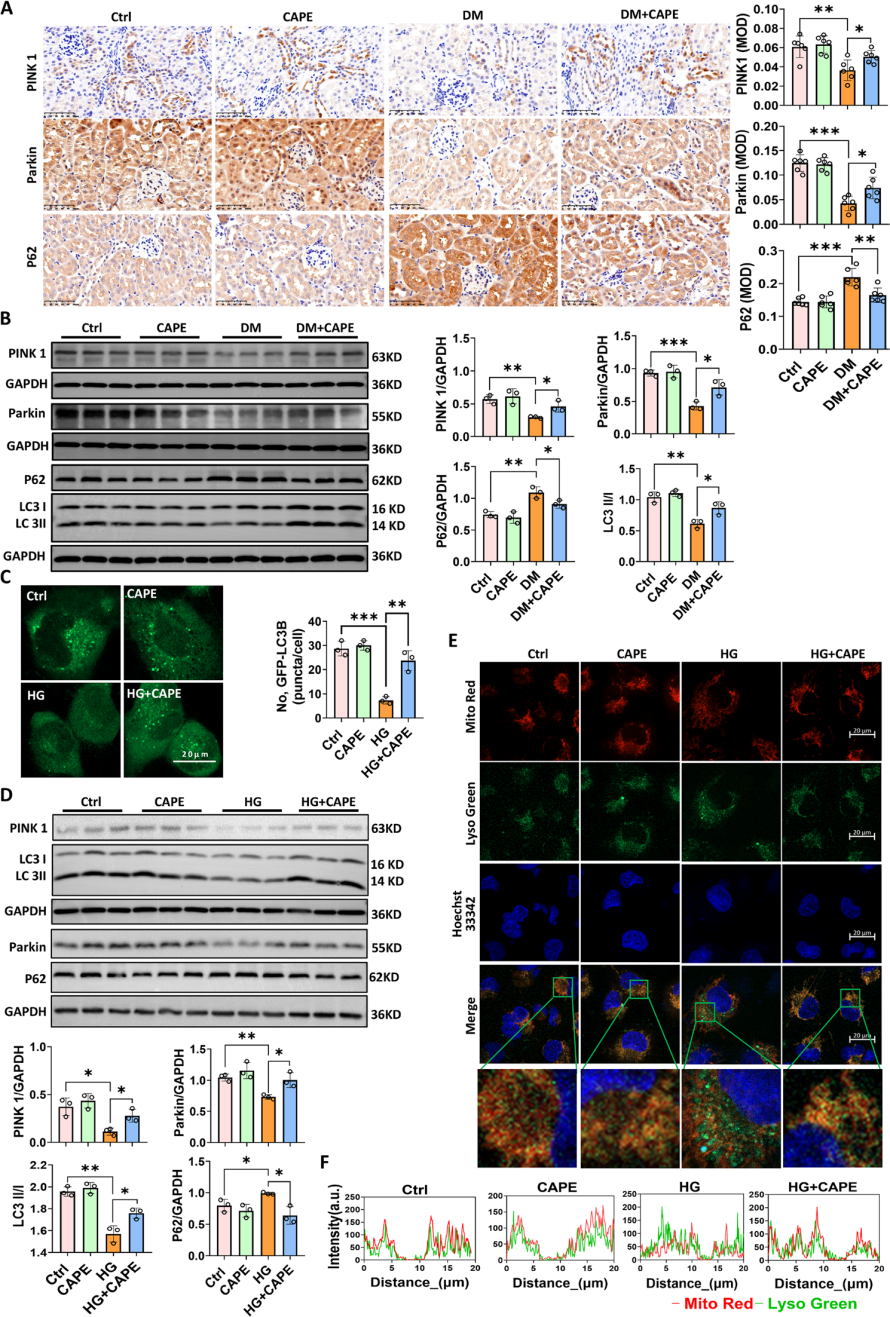
CAPE rescued the renal tubular mitophagy in vivo and in vitro. **A** Immunohistochemical staining of mitophagy-related proteins (PINK1, Parkin, P62) in renal tissues of different mice (Magnification, ×400). PINK1 and Parkin were significantly reduced in diabetic mice, a reduction partially rescued by CAPE treatment. **B** Western blot analysis of PINK1, Parkin, P62, and LC3 in kidney tissues. Diabetic mice exhibited decreased PINK1 and Parkin levels, a reduced LC3-II/LC3-I ratio, and elevated P62 levels, all of which were partially reversed by CAPE. All data are expressed as mean ± SD (*n* = 6). **C** Autophagy intensity in HK-2 cells was assessed by Ad-GFP-LC3B puncta formation (scale bar = 20 μm). HG exposure (24 h) significantly reduced LC3B puncta in HK-2 cells, and this effect was significantly reversed by CAPE intervention. **D** Protein levels of mitophagy markers (PINK1, Parkin, P62, LC3B) in HK-2 cells were analyzed. HG decreased PINK1, Parkin, and the LC3B II/I ratio, which were reversed by CAPE. **E** Colocalization of mitochondria (MitoTracker Red) and lysosomes (LysoTracker Green) in HK-2 cells was observed (scale bar = 20 μm). HG impaired Colocalization, which was partially rescued by CAPE. **F** Colocalization analysis using Image J software revealed that there was an asynchronous change in red and green fluorescence intensity in HG-stimulated HK-2 cells, indicating impaired colocalization between mitochondria and lysosomes. CAPE was able to partially reverse the inhibition of Colocalization of mitochondria and lysosomes by HG. All data are expressed as mean ± SD (*n* = 3). ^*^P < 0.05; ^**^P < 0.01; ^***^P < 0.001

We compared the expression levels of PINK1 in the renal tubules between the DKD patient and the control group with para-cancerous tissues. The results, as vividly illustrated in Supplementary Fig. 2, clearly demonstrated a significant reduction of PINK1 in the renal tubules of patients with DKD. Moreover, PINK1 decreased as the eGFR declined. Immunohistochemical staining revealed a notable decrease in PINK1 and Parkin, key indicators of mitophagy, in the renal tubules of DM mice (Fig. 6A). In comparison to the untreated diabetic mice, the diabetic mice treated with CAPE showed an increase in PINK1 and Parkin in the renal tubules **(**Fig. 6A). Western blotting results corroborated the immunohistochemical findings (Fig. 6B).

To evaluate autophagy intensity, we employed the Ad-GFP-LC3B single fluorescence autophagy indicator. Under normal conditions, HK-2 cells showed moderate intensity punctate green fluorescence, representing physiological levels of autophagy. Under HG conditions, there was a notable decrease in the LC3B puncta, which was reversed by CAPE (Fig. 6C). Immunoblotting results showed that the levels of the LC3B II/I ratio, PINK1, and Parkin were decreased in HG - cultured HK −2 cells (Fig. 6D). CAPE (5 µM) treatment increased PINK1 and Parkin levels (Fig. 6D).

However, increases in LC3B puncta, the LC3B II/I ratio, PINK1 levels, and Parkin levels do not necessarily indicate enhanced mitophagy. It might also be that the downstream of mitophagy is impeded. To further evaluate the downstream of mitophagy, we identified the colocalization of lysosomes and mitochondria and detected p62. The findings indicated that HG significantly increased the level of p62 and decreased the colocalization of LysoTracker Green and MitoTracker Red. These effects were reversed by CAPE, indicating that CAPE induced mitophagolysosome formation (Fig. 6D-F). The results indicate that CAPE has the potential to mitigate the inhibition of mitophagy initiation induced by HG and maintain downstream mitophagic flux.

### CAPE interacted with PINK1 as its specific target

**Fig. 7:**
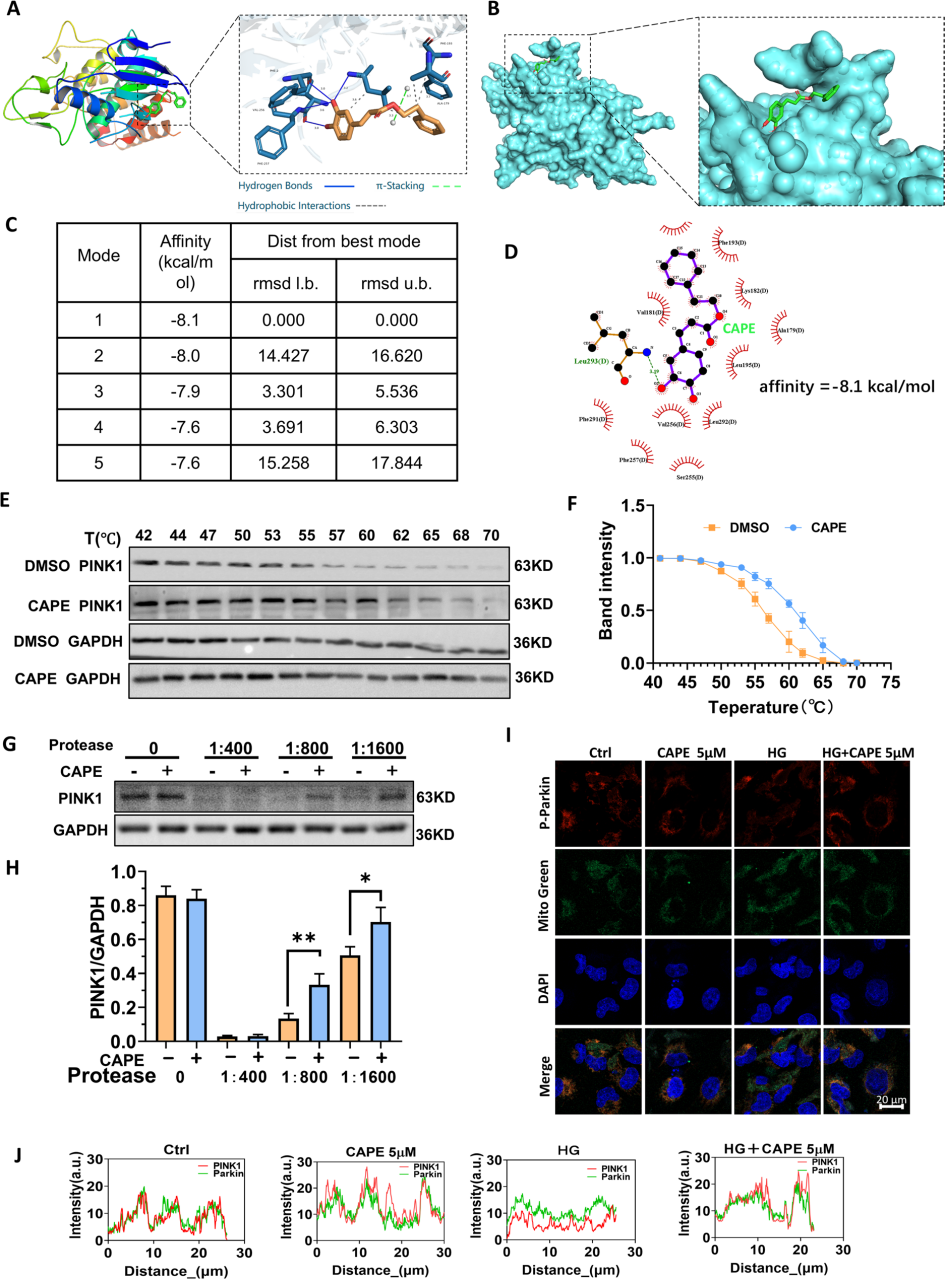
CAPE interacted with PINK1 as its specific target. **A** Three-dimensional structure of CAPE-PINK1 molecular docking. **B** CAPE is located within the molecular pocket of PINK1 protein. **C** Alignment of lowest binding energy for molecular docking of CAPE-PINK1. **D** Two-dimensional interaction diagram showing hydrogen bonds, π-π stacking, and hydrophobic interactions between CAPE and PINK1 residues. **E **and** F** HK-2 cells were incubated with DMSO or CAPE for 1 h, and then denatured at different temperatures. The PINK1 protein was detected by Western blot, and a curve graph was plotted for semi-quantitative analysis. CAPE increased PINK1 melting temperature from 56.51 °C to 62.72 °C. **G **and** H** The Drug Affinity Responsive Target Stability (DARTS) and the semi-quantitative histogram of PINK1. CAPE (200 µM) protected PINK1 from thermolysin degradation in HK-2 cell lysates. **I** Colocalization of phosphorylated Parkin (Ser65) and mitochondria (Mito-Tracker Green) in HK-2 cells (scale bar = 20 μm). **J** Colocalization analysis of phosphorylated Parkin and Mito-Tracker Green was performed using Image J software. All data are expressed as mean ± SD (*n* = 3). ^*^P < 0.05; ^**^P < 0.01

The mitophagy-associated proteins PINK1 have a pivotal effect on maintaining mitophagy [47]. The molecular docking results revealed that CAPE bound to the PINK1 active site with the lowest binding energy of −8.1 kcal/mol (Fig. 7A-D). Hydrogen bonds, π-π interactions, and hydrophobic interactions are linked between CAPE and amino acid residues in the PINK1 protein (Fig. 7A and D). Specifically, two phenolic hydroxyl groups of the CAPE side chain form four hydrogen bonds with amino acids VAL-256, PHE-257, PHE-291, and LEU-293 (Fig. 7A). The benzene ring on the outer side interacts through an π-π interaction with the amino acid residue PHE-193 (Fig. 7A). CAPE is located within the molecular pocket of PINK1 protein. Additionally, the benzene rings on both sides engage in hydrophobic interactions with PHE-193, ALA-179, and LEU-293 (Fig. 7A and D). These forces lead to the formation of a stable complex between CAPE and PINK1. The binding energies of the top five docking results are all less than − 7.5 kcal/mol (Fig. 7C). The results revealed that CAPE demonstrates the potential to engage in more diverse interactions with the PINK1 protein.

We further employed the Cellular Thermal Shift Assay (CETSA) and the Drug Affinity Responsive Target Stability (DARTS) assay to confirm that CAPE could specifically bind to PINK1. After the addition of CAPE, the thermal denaturation curve of the protein shifted to the right, and the melting temperature increased from 56.51 °C to 62.72 °C (Fig. 7E and F), suggesting that CAPE enhanced the thermal stability of PINK1 within a specific temperature range. In DARTS the total cellular proteins were incubated with CAPE (200 µM) and different concentrations of thermolysin (Fig. 7G and H). The results showed that CAPE protected PINK1 from proteolytic degradation. Subsequently, we evaluated the activity of PINK1 by detecting the phosphorylation level of Parkin. PINK1 can promote the phosphorylation of Parkin at the Ser65 site. Immunofluorescence staining found that CAPE promoted the phosphorylation of Parkin and increased its colocalization with mitochondria (Fig. 7I and J). These findings indicate that CAPE interacted with PINK1, and PINK1 was its specific target.

The presence of the α, β-unsaturated ketone moiety in the structure of CAPE allows it with the potential to target and activate nuclear factor E2-related factor 2 (Nrf2). Therefore, we investigated the interaction between CAPE and Nrf2. The results are presented in Supplementary Fig. 3. The binding energy of only one docking site was lower than − 7.0 kcal/mol when CAPE was docked with Nrf2. The results of the DARTS assay indicated that CAPE did not enhance the stability of Nrf2 against proteases. In addition, in HK-2 cells treated with high glucose for 12 h, CAPE did not significantly promote the translocation of Nrf2 to the nucleus, and specifically blocking the Nrf2 pathway with ML385 did not prevent CAPE from upregulating GPX4 (Supplementary Fig. 3E-G).

### CAPE restored mitophagy defects via the PINK1-dependent pathway

**Fig. 8:**
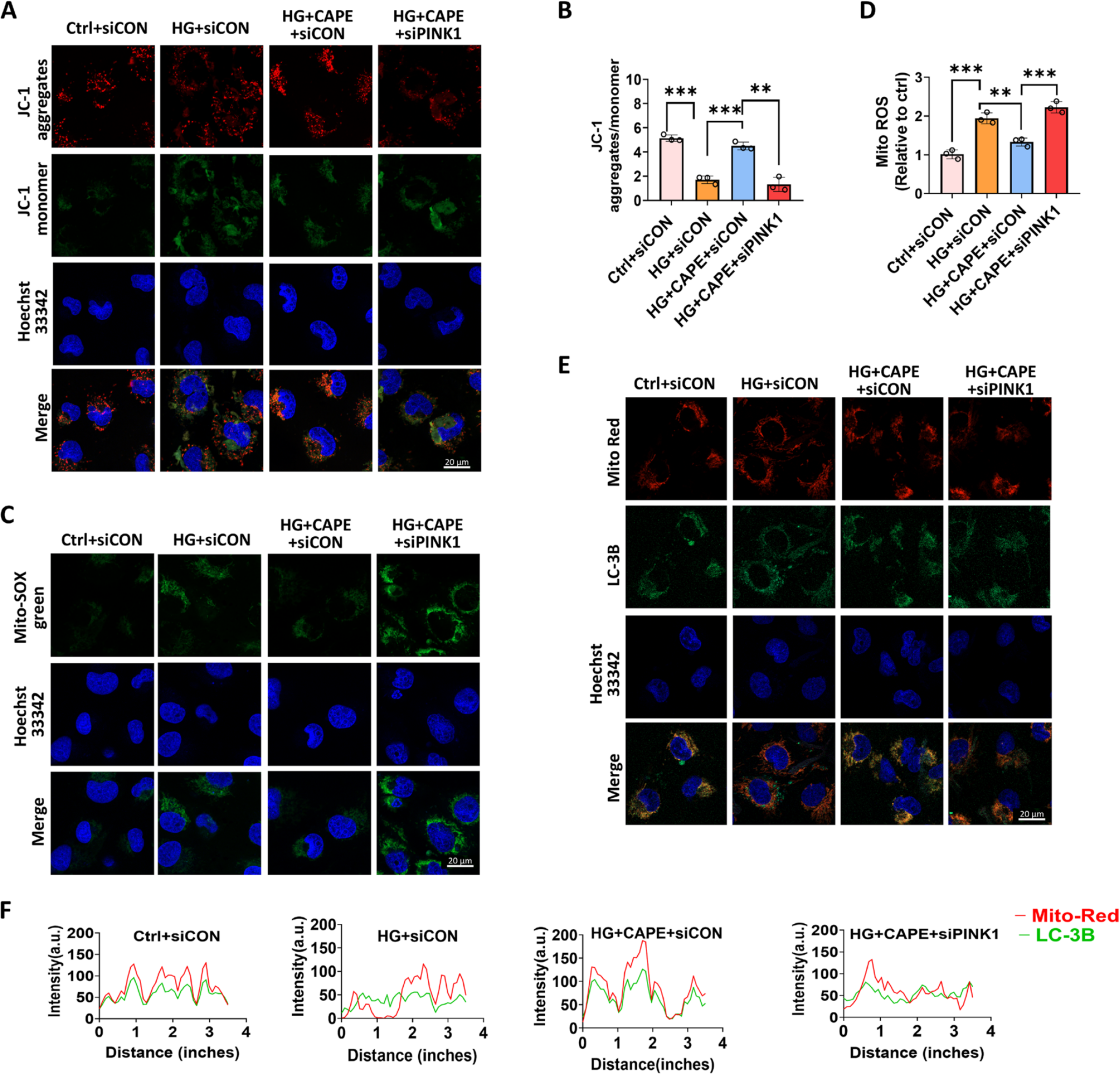
CAPE restored mitophagy defects via the PINK1-dependent pathway. **A **and **B** JC-1 staining of mitochondrial membrane potential (MMP) in HK-2 cells. HG induced MMP depolarization (increased green fluorescence, decreased red fluorescence), which was reversed by CAPE but partially blocked by PINK1 siRNA (scale bar = 20 μm). **C **and** D** Mitochondrial ROS in HK-2 cells detected by MitoSOX Green. CAPE suppressed HG-induced ROS overproduction, an effect partially neutralized by PINK1 siRNA (scale bar = 20 μm). **E** Confocal images showing colocalization of LC3B (green) and mitochondria (Mito-Tracker Red, red) in HK-2 cells (scale bar = 20 μm). **F** Colocalization analysis of LC3B with Mito-Tracker Red using Image J software. The results showed that the changes in red fluorescence and green fluorescence intensity in the HG group were not synchronized, indicating that HG hindered the colocalization of LC3B and mitochondria in HK-2 cells. The effects of CAPE on the colocalization of LC3B with mitochondria were significantly neutralized by PINK1 siRNA. All data are expressed as mean ± SD (*n* = 3). ^**^P < 0.01; ^***^P < 0.001

To confirm whether CAPE restored mitophagy via the PINK1-dependent pathway, we used siRNA to silence PINK1. Fluorescence microscopy revealed a significant increase in the green intensity of JC-1 accompanied by a notable decrease in its red intensity when cells were exposed to HG (Fig. 8A and B). CAPE restored the MMP, but these effects were partially neutralized by the siRNA targeting PINK1 (Fig. 8A and B). Additionally, although CAPE reversed HG-induced mitochondrial ROS overproduction, the beneficial effects of CAPE were partially blocked by the siRNA targeting PINK1 (Fig. 8C and D). Further results showed that PINK1 siRNA significantly counteracted the effects of CAPE on the colocalization of LC3B with mitochondria (Fig. 8E and F). These results revealed that CAPE may restore HG-induced mitophagy defects in TECs via the PINK1-dependent pathway.

### CAPE mitigated HG-induced ferroptosis via the PINK1-dependent mitophagy

**Fig. 9:**
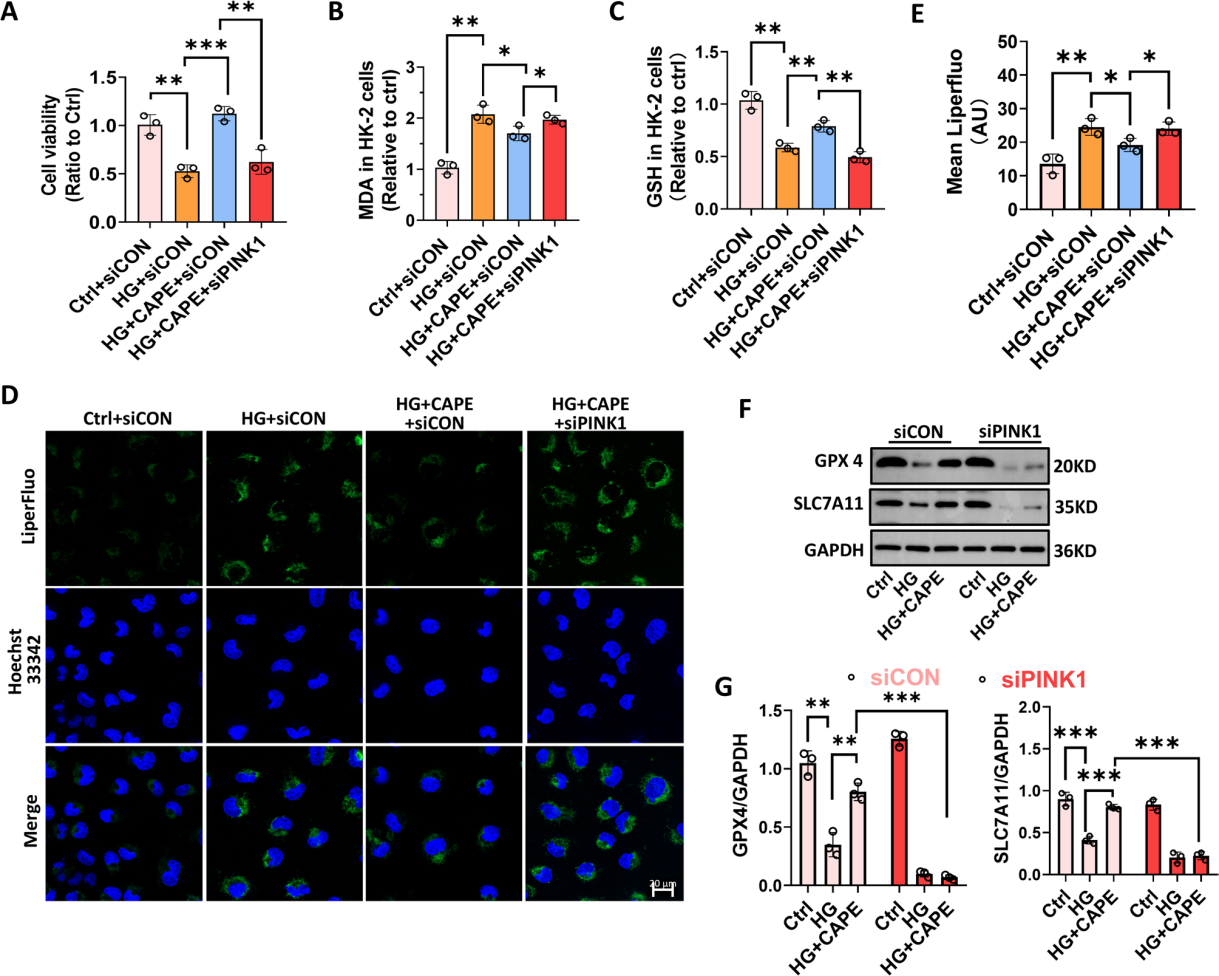
CAPE mitigated HG-induced ferroptosis via PINK1-dependent mitophagy. **A** Cell viability of HK-2 cells was measured by CCK-8 assay. CAPE alleviated HG-induced cell death at 24 h, and this effect was significantly blocked by PINK1 siRNA. **B** and **C** MDA and GSH levels in HK-2 cells. CAPE suppressed HG-induced MDA elevation and GSH reduction at 24 h, effects that were partially counteracted by PINK1 siRNA. **D** and **E** Fluorescence imaging of lipid hydroperoxides in HK-2 cells by Liperfluo staining. CAPE reduced HG-induced lipid peroxidation at 48 h, an effect reversed by PINK1 siRNA (scale bar = 20 μm). **F** and **G** Immunoblotting of GPX4 and SLC7A11 in HK-2 cells. CAPE upregulated both proteins in HG-stimulated HK-2 cells at 48 h, and these effects were abolished by PINK1 siRNA. All data are expressed as mean ± SD (*n* = 3). ^*^P < 0.05; ^**^P < 0.01; ^***^P < 0.001

The measurement of cell viability showed that CAPE alleviated HG-caused cell death, but the beneficial effect was significantly blocked by knocking down PINK1 using siRNA (Fig. 9A). Treatment with CAPE inhibited the excessive lipid hydroperoxides induced by HG, while increasing the levels of GSH (Fig. 9B-E). However, silencing PINK1 partially counteracted the effects of CAPE (Fig. 9B-E), suggesting that CAPE mitigated lipid peroxidation via the PINK1-dependent pathway. Western blot analysis revealed that CAPE treatment upregulated GPX4 and SLC7A11 in HG-stimulated cells (Fig. 9F and G). However, silencing PINK1 partially impeded these effects of CAPE (Fig. 9F and G), indicating that CAPE attenuated ferroptosis in HG-challenged renal TECs via the PINK1-dependent pathway.

## Discussion

This study demonstrated that ferroptosis in tubular epithelial cells (TECs) is involved in the development of diabetic kidney disease (DKD), which is evidenced by the following phenomena: In diabetic mice and the TECs stimulated by HG, GPX4 and SLC7A11 decreased. Meanwhile, the free iron increased, the antioxidant capacity declined, and lipid peroxidation products accumulated. In addition, the mitophagy-related protein PINK1 significantly decreased, leading to defective mitophagy. In contrast, CAPE interacted with PINK1 as its specific target to rescue the defective mitophagy, significantly alleviating tubular ferroptosis, and mitigating DKD. Furthermore, our findings showed that knocking down PINK1 partially abolished the effect of CAPE against ferroptosis. These results indicate that CAPE protects renal tubular cells from diabetes-induced ferroptosis by rescuing PINK1-dependent mitophagy.

While glomerular pathology has traditionally been the focus of DKD [48], it is now widely realized that tubulopathy also exerts a pivotal role [49, 50]. In TECs, HG can trigger the overproduction of ROS and initiate the death pathways, contributing to the progression of DKD [51, 52]. Although the exact molecular mechanisms involved in these phenomena remain unclear, prior research has indicated that tubular ferroptosis participates in the advancement of DKD [9–11]. Ferroptosis is a type of cell death that arises as lipid peroxidation surpasses the antioxidant capacity of GPX4 [53]. Recent evidence elucidated that ferroptosis plays a pivotal role in the progression of renal fibrosis by driving tubular epithelial cell injury through lipid peroxidation and GSH depletion, thereby accelerating the transition from acute kidney injury to fibrotic remodeling[54]. Experimental evidence from unilateral ureteral obstruction (UUO) and 5/6 nephrectomy models demonstrates that ferroptosis inhibitors, such as liproxstatin-1 and tectorigenin, significantly attenuate fibrosis by suppressing lipid peroxidation and collagen deposition, highlighting their therapeutic potential [55–58]. Mechanistically, ferroptosis activates pro-fibrotic signaling pathways (e.g., TGF-β/Smad3), thereby promoting extracellular matrix deposition and renal interstitial fibrosis [57, 58]. In the context of diabetes mellitus, inhibition of ferroptosis has also been shown to alleviate tubular injury in DKD and slow down its progression [9, 12, 13, 59]. Consistent with previous studies, in DKD model mice, we observed a marked downregulation of GPX4 and SLC7A11 accompanied by upregulated TFR1 expression compared to non-diabetic controls. These alterations were associated with enhanced lipid peroxidation, collectively suggesting ferroptosis as a key contributor to tubular cell death in DKD.

CAPE, derived from propolis, has been identified as a potential antioxidant compound [60]. While prior studies by Wang X et al. revealed that CAPE and its derivatives ameliorate DKD by attenuating oxidative stress [61], no evidence has linked its nephroprotective effects to ferroptosis. Notably, our study is the first to demonstrate that CAPE significantly upregulates renal GPX4 and SLC7A11 expression while downregulating TFR1 and reducing iron overload in DKD mice compared to non-diabetic controls. Crucially, these molecular adjustments effectively attenuated lipid peroxidation, suppressed renal interstitial fibrosis, and inhibited tubular epithelial cell transdifferentiation. However, the precise mechanisms underlying CAPE-mediated regulation of ferroptosis in TECs remain unclear. Further elucidation of these mechanisms may open new therapeutic avenues for halting DKD progression.

We collected 499 target genes of CAPE and 564 target genes related to ferroptosis from multiple databases, and then identified 31 intersection targets. Enrichment analysis showed that these intersection targets are in mitochondria and play a role in regulating mitophagy and oxidative stress. Previous studies have also suggested that mitochondria are pivotal in numerous regulated cell demise mechanisms [62]. Mitochondrial iron, accounting for 20–50% of cellular iron [15], participates in ferroptosis induced by cystine depletion, which leads to the buildup of lipid peroxidation products [63]. Consistent with this, the ferroptosis inducer erastin has been demonstrated to enhance mitochondrial ROS production during the ferroptosis process, leading to mitochondrial membrane depolarization and ATP depletion [64]. Renal TECs have high levels of mitochondria, rendering them particularly susceptible to mitochondrial malfunction [20, 65]. Mitophagy is essential for removing dysfunctional mitochondria and regulating oxidative damage [23, 66]. Studies have demonstrated mitophagy suppresses renal fibrosis progression by degrading damaged mitochondria, thereby reducing ROS generation [30, 67]. Activation of mitophagy alleviates renal fibrosis by improving mitochondrial function and inhibiting the TGF-β1/Smad pathway, as validated in the UUO model [68, 69]. Similarly, chronic hypoxia significantly inhibits the expression of epithelial-mesenchymal transition (EMT) and fibrosis markers (such as α-SMA, Collagen I, and Fibronectin) in yak renal TECs by activating mitophagy [70]. Notably, the PINK1 pathway and SIRT1-PINK1 axis have been identified as potential therapeutic targets for renal fibrosis by restoring mitochondrial homeostasis [71, 72]. Recently, extensive discussion has been centered on the impact of mitophagy on DKD [19, 20, 73]. Mitochondria-targeted antioxidants were demonstrated to restore mitophagy defects in DKD models and protect TECs, podocytes, and M1/M2 macrophage phenotypes [19, 27, 74]. The PINK1-mediated mitophagy prevents apoptosis of renal TECs triggered by HG, thereby mitigating DKD [75, 76]. In this study, we found that PINK1 was significantly reduced in DKD patients, and the reduction was associated with declining eGFR. In untreated diabetic mice, PINK1 and Parkin decreased. In vitro, HG reduced autophagy-related markers such as LC3B puncta, the LC3B II/I ratio, PINK1 and Parkin, hinting that HG inhibited the initiation of mitophagy. To assess mitophagy downstream, we examined lysosome-mitochondria colocalization and p62 levels. HG increased p62 levels and decreased colocalization, while CAPE reversed these effects, suggesting CAPE promotes mitophagolysosome formation. Overall, our study demonstrates that CAPE can mitigate HG-induced inhibition of mitophagy initiation and maintain mitophagic flux.

We further elucidated the precise mechanisms underlying CAPE-mediated mitophagy in TECs under DKD condition. Although the α, β-unsaturated ketone moiety in its structure of CAPE allows it to interact with Nrf2, the binding energy of only one binding site is lower than − 7.0 kcal/mol. The DARTS assay indicated that CAPE did not enhance the stability of Nrf2 against proteases. In addition, blocking the Nrf2 pathway with ML385, a specific Nrf2 inhibitor, did not inhibit the upregulation of GPX4 expression by CAPE. Therefore, our current findings do not support the hypothesis that CAPE inhibits HG-induced ferroptosis in renal TECs via the Nrf2 pathway. Conversely, molecular docking simulation experiments suggested that CAPE is steadily bound to the PINK1 active pocket, enhancing the thermal stability of the PINK1 protein and protecting the PINK1 protein from degradation by proteolytic enzymes. The effects of CAPE on rescuing mitophagy were significantly neutralized by PINK1 siRNA. These results confirm that CAPE interacted with PINK1, and PINK1 was its specific target.

Previous studies have revealed a close interplay between mitophagy and ferroptosis through oxidative stress and iron metabolism [70, 77]. Our current study further demonstrated that PINK1 knockdown partially abolished the protective effect of CAPE against ferroptosis, suggesting that CAPE rescues renal tubular cells ferroptosis by interacting with PINK1 as its specific target thereby restoring mitophagy. However, further exploration of the regulatory mechanisms linking mitophagy to ferroptosis, as well as their synergistic or antagonistic roles in DKD, may provide novel therapeutic strategies targeting the mitophagy-ferroptosis axis.

## Conclusion

**Fig. 10:**
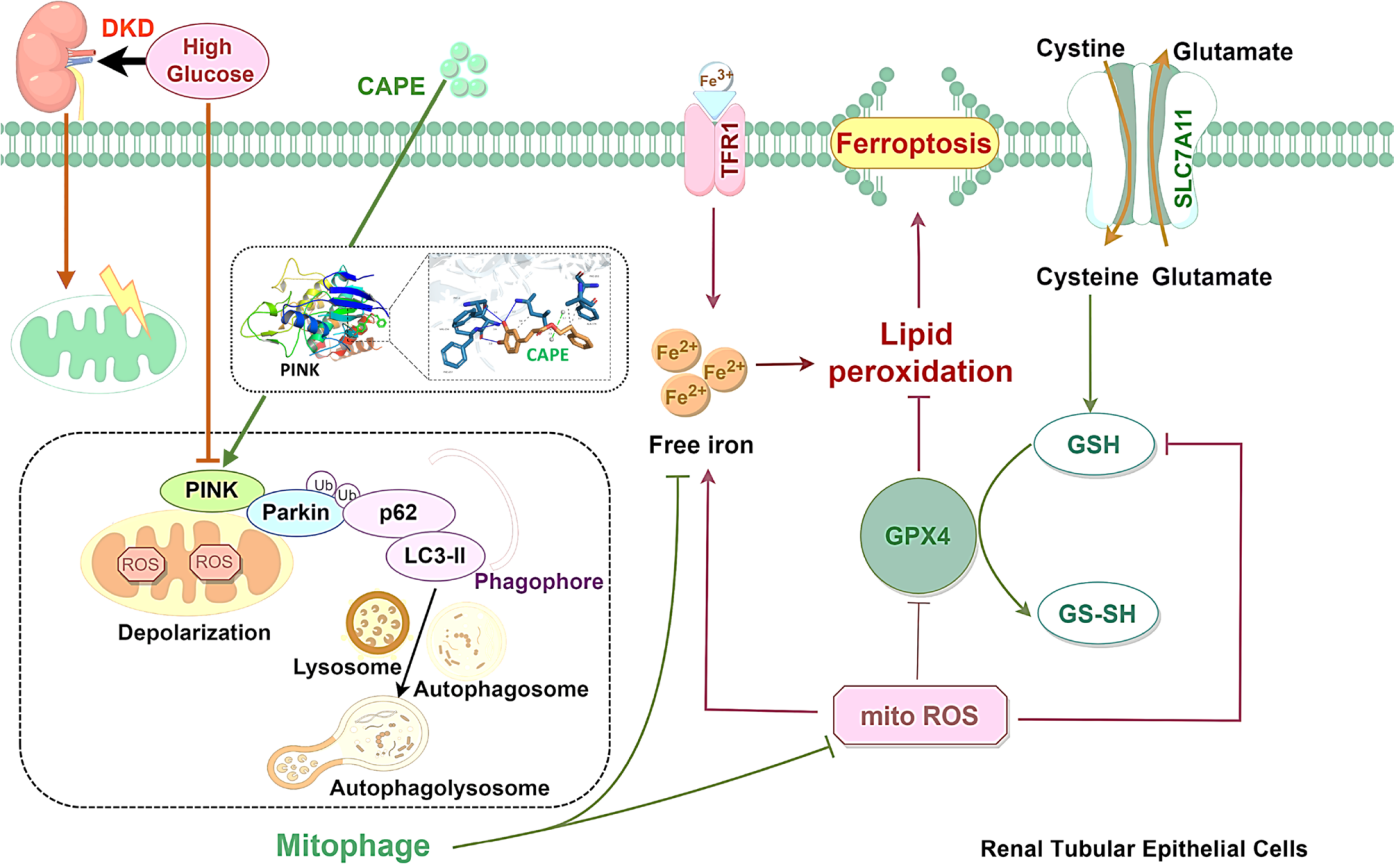
Diagram of specific mechanism of action in this study. HG induces mitochondrial damage in renal tubular epithelial cells while concurrently suppressing PINK1-mediated mitophagy, thus triggering the accumulation of dysfunctional mitochondria. CAPE directly binds to the active site of PINK1 and interacts with PINK1 protein, thereby up-regulating PINK1-dependent mitophagy pathway, subsequently reducing mitochondrial ROS production and intracellular iron overload. The reconstituted mitophagy downregulates ferroptosis markers (e.g., TFR1) and upregulates antioxidant defense systems (e.g., GPX4/SLC7A11), thereby alleviating lipid peroxidation damage

The present research demonstrated that CAPE has potential therapeutic benefits for DKD by alleviating ferroptosis. Specifically, CAPE rescues PINK1-mediated mitophagy, thereby protecting renal TECs against ferroptosis (A schematic diagram was shown in Fig. 10). These findings hint that CAPE shows potential as a therapeutic agent to prevent tubular injury in DKD.

### Limitations of the study

Limitations of the study include that our model only simulated one specific DKD model. We plan to conduct future studies using more extended DKD models, observing the long-term administration of CAPE. Furthermore, we plan to employ conditional PINK1 knockout mice, high-throughput sequencing, proteomics, and other advanced technologies in future research to further elucidate the specific molecular mechanism of CAPE in DKD.

## Supplementary Information

Supplementary Material 1.

## Data Availability

No datasets were generated or analysed during the current study.
