# Supplementary material for: Caffeic acid phenethyl ester protects renal tubular epithelial cells against ferroptosis in diabetic kidney disease via restoring PINK1-mediated mitophagy
Source: Mol Med. 2025 Jul 24;31:264. doi: 10.1186/s10020-025-01318-y (PMC12288367; doi:10.1186/s10020-025-01318-y)
Supplement: Supplementary file 1 — Supplementary Material 1. [file 10020_2025_1318_MOESM1_ESM.docx]

**Supplementary Figure**


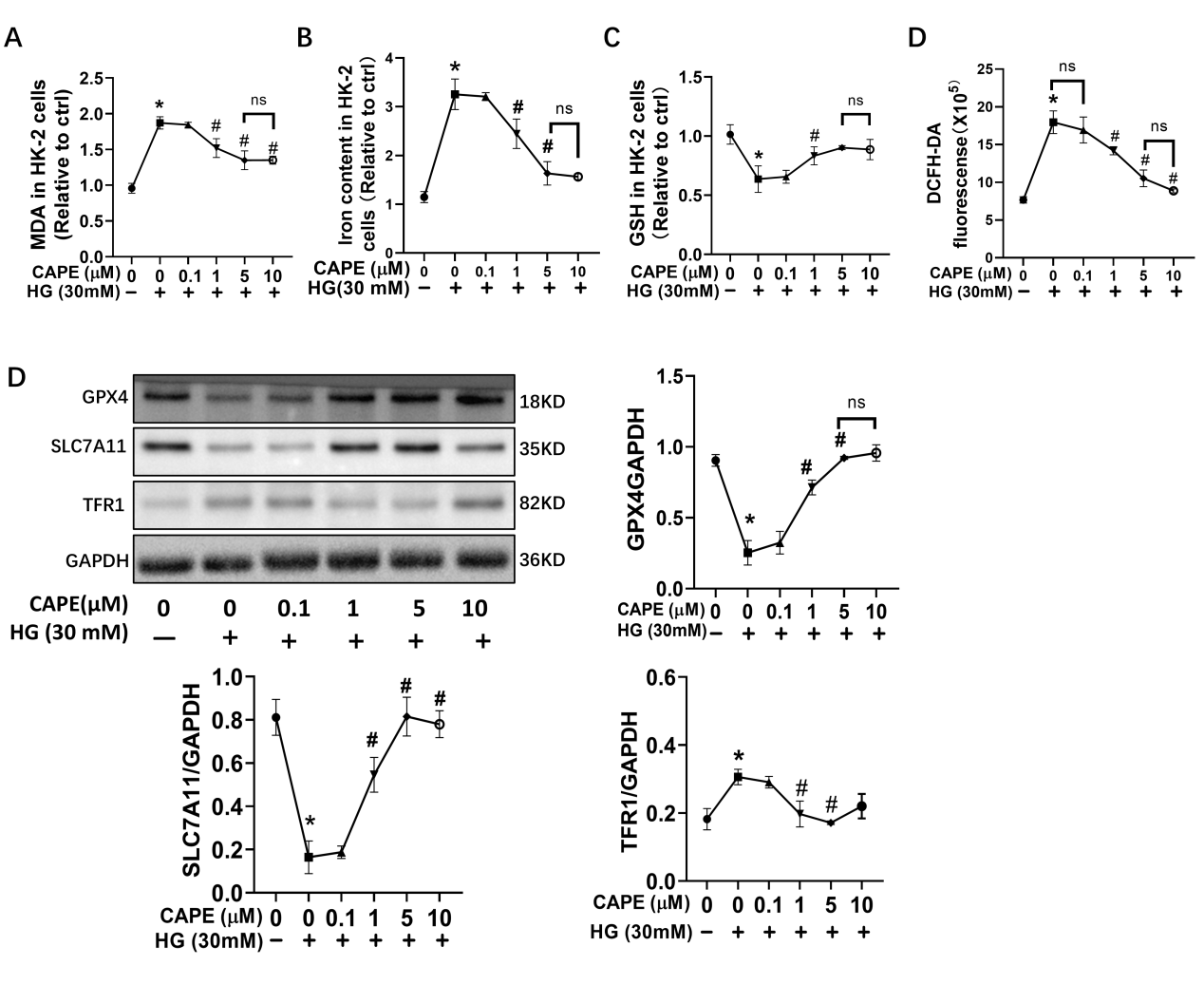


**Supplementary Figure 1 The effects of different doses of CAPE on ferroptosis of HK-2 cells induced by high glucose (HG).** The results revealed that within the concentration range of 0.1–5 μM, as the CAPE dose increased, the inhibitory effect on ferroptosis also increased. However, when compared to the 5 μM dose, the 10 μM dose did not exhibit a distinct advantage in terms of inhibiting ferroptosis. ***, compared with the control group, **p* < 0.05; #, compared with the HG group, #*p* < 0.05; ns, no significance.


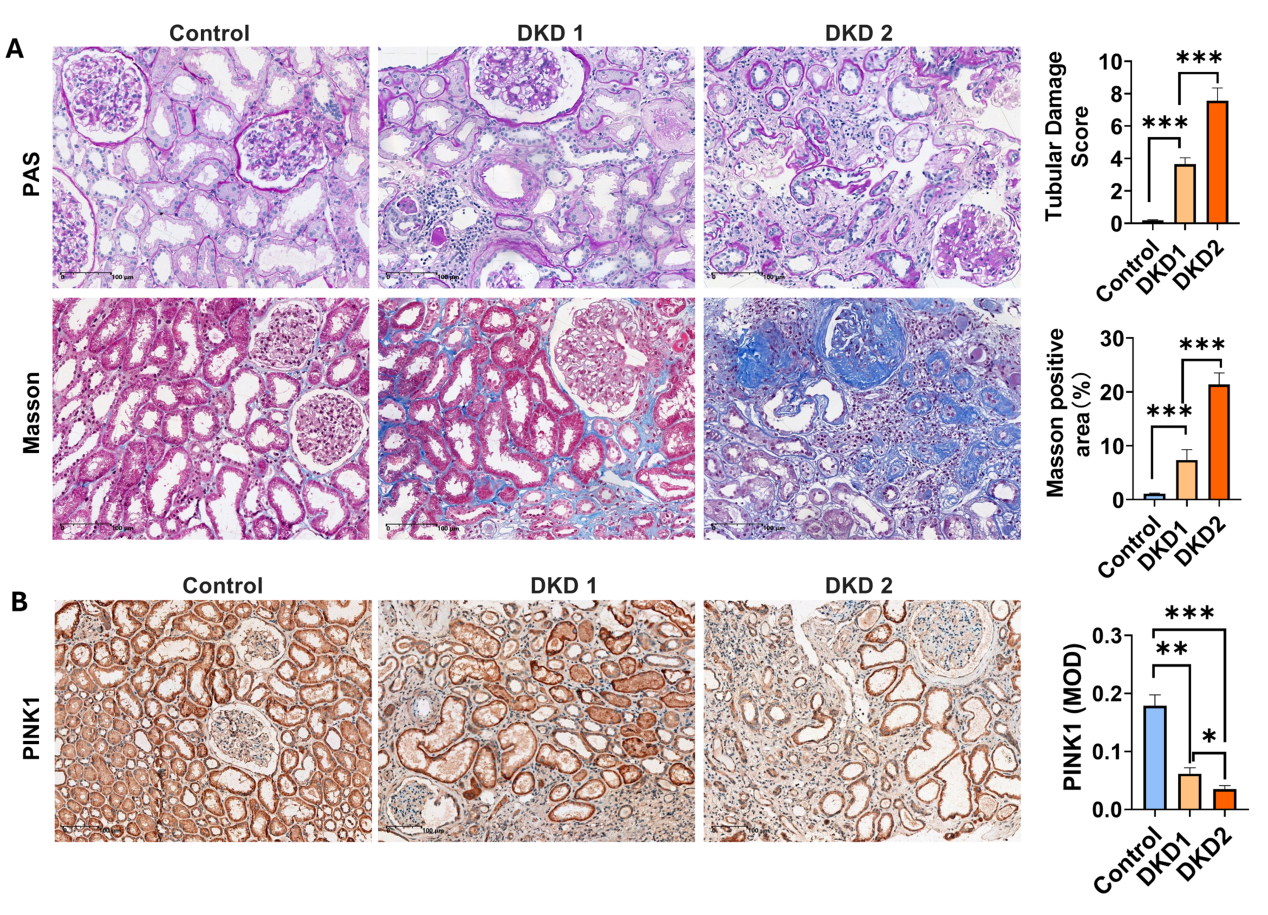


**Supplementary Figure 2 The expression of PINK1 in the kidneys of patients was detected by immunohistochemistry. (A)** Periodic acid-Schiff (PAS) and Masson's trichrome staining of renal tissue. **(B)** Immunohistochemical detection of PINK1 expression in renal tissue. The results demonstrated a significant reduction of PINK1 in the renal tubules of patients with DKD. Moreover, PINK1 decreased as the eGFR declined. **Control**, Para-cancerous renal tissues; **DKD1**, DKD patients with GFR ≥ 60ml/min; **DKD2**, DKD patients with eGFR＜60ml/min; Control, Para-cancerous renal tissues (scale bar = 100 μm).  *^*^P* <0.05; *^**^P* < 0.01; *^***^P* < 0.001.


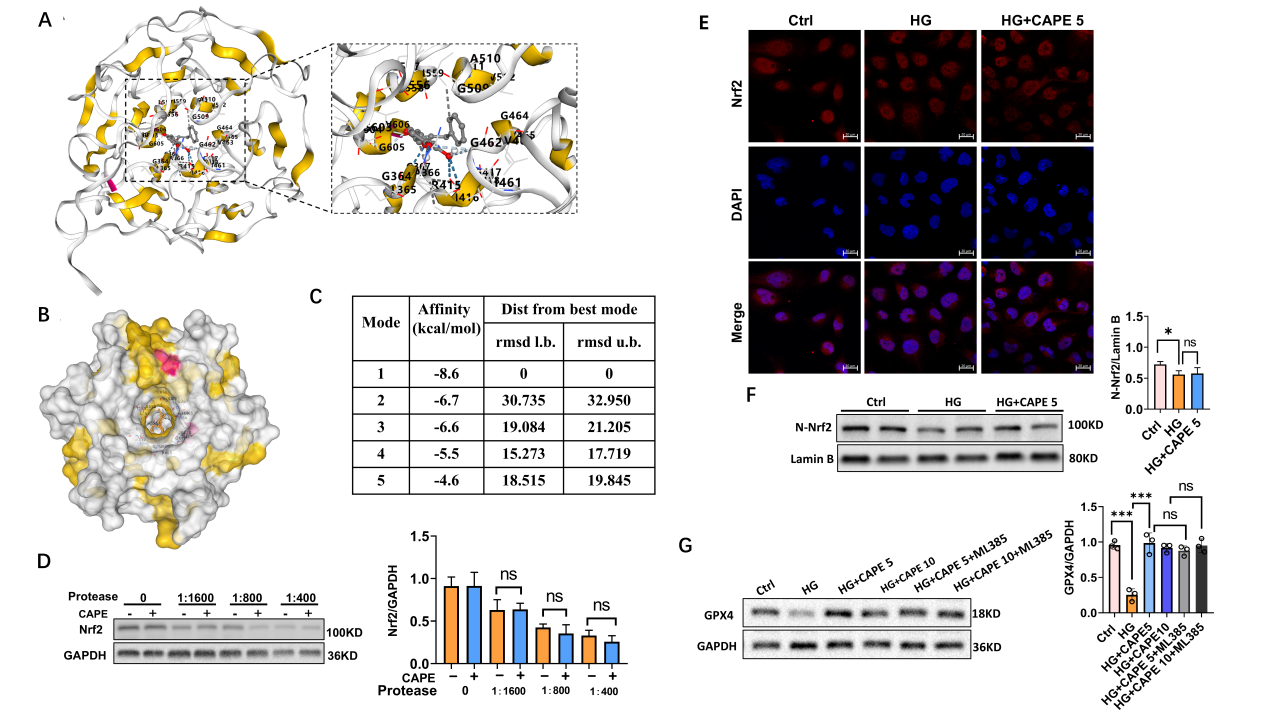


**Supplementary Figure 3 The impact of CAPE on the Nrf2 pathway in HG-treated HK-2 cells. (A**–**C)** Molecular docking of CAPE with Nrf2 showed only one binding site with energy < -7.0 kcal/mol. **(D)** The DARTS assay indicated that CAPE did not enhance the stability of Nrf2 against proteases in HK-2 cells. **(E–F)** The cellular immunofluorescence and immunoblotting showed that CAPE did not significantly promote the nuclear translocation of Nrf2 in HG-treated HK-2 cells for 12 hours (scale bar = 20 μm). **(G)** ML385, a specific inhibitor of Nrf2, failed to block CAPE-induced GPX4 upregulation in HK-2 cells, suggesting that CAPE alleviates ferroptosis not through the Nrf2 pathway. *^*^P* < 0.05; *^***^P* < 0.001; ns, no signific
